# An integrated imaging sensor for aberration-corrected 3D photography

**DOI:** 10.1038/s41586-022-05306-8

**Published:** 2022-10-19

**Authors:** Jiamin Wu, Yuduo Guo, Chao Deng, Anke Zhang, Hui Qiao, Zhi Lu, Jiachen Xie, Lu Fang, Qionghai Dai

**Affiliations:** 1 Department of Automation, Tsinghua University, Beijing, China; 2 Beijing National Research Center for Information Science and Technology, Tsinghua University, Beijing, China; 3 Institute for Brain and Cognitive Sciences, Tsinghua University, Beijing, China; 4 Department of Electronic Engineering, Tsinghua University, Beijing, China; 5 Tsinghua Shenzhen International Graduate School, Tsinghua University, Shenzhen, China

**Keywords:** Imaging and sensing, Astronomical optics, Adaptive optics, Optical sensors, Imaging techniques

## Abstract

Planar digital image sensors facilitate broad applications in a wide range of areas^1–5^, and the number of pixels has scaled up rapidly in recent years^2,6^. However, the practical performance of imaging systems is fundamentally limited by spatially nonuniform optical aberrations originating from imperfect lenses or environmental disturbances^7,8^. Here we propose an integrated scanning light-field imaging sensor, termed a meta-imaging sensor, to achieve high-speed aberration-corrected three-dimensional photography for universal applications without additional hardware modifications. Instead of directly detecting a two-dimensional intensity projection, the meta-imaging sensor captures extra-fine four-dimensional light-field distributions through a vibrating coded microlens array, enabling flexible and precise synthesis of complex-field-modulated images in post-processing. Using the sensor, we achieve high-performance photography up to a gigapixel with a single spherical lens without a data prior, leading to orders-of-magnitude reductions in system capacity and costs for optical imaging. Even in the presence of dynamic atmosphere turbulence, the meta-imaging sensor enables multisite aberration correction across 1,000 arcseconds on an 80-centimetre ground-based telescope without reducing the acquisition speed, paving the way for high-resolution synoptic sky surveys. Moreover, high-density accurate depth maps can be retrieved simultaneously, facilitating diverse applications from autonomous driving to industrial inspections.

## Abstract (web summary)

A meta-imaging sensor detects an extra-fine 4D light field distribution using a vibrating microlens array, enabling high-resolution 3D photography up to a gigapixel with fast aberration correction, demonstrated on a telescope aimed at the Moon.

## Main

Two-dimensional (2D) imaging sensors have revolutionized many fields, including industrial inspection, mobile devices, autonomous driving^1^, surveillance^2^, medical diagnosis^3^, biology^4^ and astronomy^5^. Benefiting from the rapid development of semiconductor industry, the number of pixels in digital sensors has grown quickly in the past decade^2,6^. However, the practical performance of most imaging systems has reached a bottleneck set by optics instead of electronics. For example, given a gigapixel sensor, the effective pixel number of an imaging system is usually limited to the megapixel level, owing to optical aberrations originating from imperfect lenses or environmental disturbances, which cause light emitted from one point to spread out over a large region on a 2D sensor^7,8^. Meanwhile, the projection of three-dimensional (3D) scenes to a 2D plane incurs the loss of various freedoms of the light field, such as depth and local coherence. As a result, it has long been a challenge to obtain high-density depth maps with an integrated sensor^9^.

Experts in optical engineering have spent hundreds of years designing perfect imaging systems for aberration correction with multiple precision-engineered lenses in a sequential mode^10^. However, the difficulty of optical design and fabrication increases exponentially with the space–bandwidth product, which describes the total number of degrees of freedoms for an optical system and sets an upper boundary on the effective pixel number due to diffraction limits^11^. In this case, high-performance incoherent imaging systems with large effective space–bandwidth products are usually very expensive and bulky, such as large-aperture telescopes^12^ and mesoscopes^13,14^. Metalens and free-form optics may potentially alleviate this problem by fabricating optimized lens surfaces when given sufficient machining precision across a large scale^15,16^. Image deblurring algorithms may improve the image contrast via accurate estimations of the point spread function (PSF)^17–19^. PSF engineering with a coded aperture preserves more information by reducing the nulls in the frequency domain^20,21^. However, it is very difficult to retrieve the high-frequency information lost by a low modulation transfer function (MTF), and these approaches usually require specific data priors and precise PSF estimations, especially for spatially nonuniform aberrations^22^. Moreover, all these methods are still sensitive to dynamic environmental aberrations with small depths of field.

Adaptive optics achieves active aberration corrections with a deformable mirror array or spatial light modulator to direct the rays emitted from one point to the same position on the sensor at different angles^5,23^. Aberrated wavefronts can be measured by a guide star and a wavefront sensor or by iterative updates according to specific evaluation metrics^24^. Adaptive optics has exhibited great success in both astronomy and microscopy and has contributed to important scientific discoveries^23^. However, the effective field of view (FOV) of the current adaptive optics approach is very small, owing to spatially nonuniform aberrations^23^. Particularly for ground-based telescopes, the aberration caused by atmospheric turbulence limits the FOV of adaptive optics to approximately 40 arcseconds in diameter, which is too small for a large synoptic survey telescope^25,26^. More importantly, current adaptive optics systems are usually complicated, bulky and expensive, which makes the development of lightweight systems or portable devices difficult.

Here we propose an integrated scanning light-field imaging framework including both hardware and software, termed a meta-imaging sensor, to achieve aberration-corrected 3D imaging with a large space–bandwidth product at low cost. Akin to metasurfaces for unprecedented manipulations of light fields with nanostructures^16^, the meta-imaging sensor facilitates measurements and syntheses of the light field in the spatial–angular domain at high speed with a vibrating coded microlens array, which are much more precise than traditional light-field techniques, decoupling the optical modulation process from data acquisition. We then achieve high-performance 3D imaging with multisite aberration correction via wave-optics-based digital adaptive optics (DAO) on a single integrated sensor. By exploiting spatiotemporal continuity, we develop an optical-flow-based motion correction algorithm to prevent motion artefacts and maintain the imaging speed (up to the camera frame rate).

To establish the capabilities of the meta-imaging sensor, we conduct quantitative analysis with diverse applications in photography, autonomous driving, industrial inspection, video surveillance and astronomy. Specifically, we obtain high-performance all-in-focus images up to gigapixel with a single lens, indicating a three-orders-of-magnitude reduction in system costs and capacity. Especially under conditions with severe nonuniform aberrations, the meta-imaging sensor achieves an over tenfold improvement in resolution. Moreover, the meta-imaging sensor facilitates multisite aberration corrections over 1,000 arcseconds in diameter on an 80-cm-aperture telescope, paving the way towards high-resolution ground-based synoptic surveys. Megapixel depth maps can be obtained simultaneously at millisecond scale with better accuracy and resolution than traditional light-field cameras for diverse industrial applications.

## Principle of the meta-imaging sensor

Traditional 2D sensors pose a great challenge on optics as a result of the long-established design philosophy for the human retina—“what you see is what you get”. Light-field imaging or plenoptic imaging provides another solution from machine vision by detecting four-dimensional (4D) spatial–angular information as building blocks and synthesizing images with arbitrary modulations during post-processing;^27,28^ this approach has shown great potential in 3D vision^29^, aberration correction^30^ and microscopy^31^. However, existing light-field cameras suffer from severely degraded spatial resolution owing to the failure of incoherent synthetic aperture (ISA), restricting their practical applications in various fields^32^. By exploiting the diffraction effect caused by the small aperture of each microlens, scanning light-field microscopy bypasses the trade-off between spatial and angular resolutions with a 2D galvo system that shifts the image plane periodically^33^, but this type of microscopy requires additional optical systems and cannot be used for universal imaging.

**Fig. 1:**
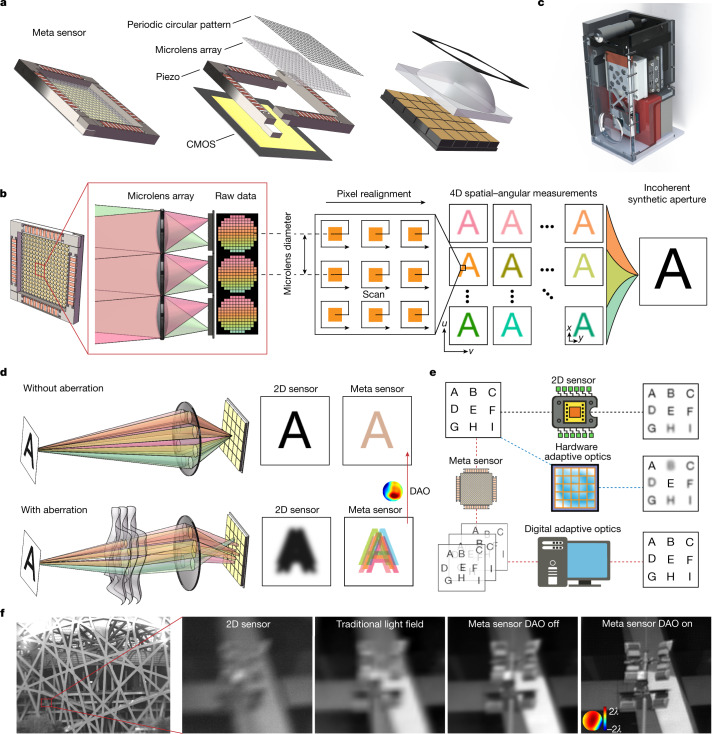
Principle of the integrated meta-imaging sensor. **a**, The meta-imaging sensor (meta sensor) integrates four main elements: a periodic circular pattern with a circular intensity mask on each microlens, a microlens array to capture the angular light distributions in every local region, a piezo stage to vibrate the microlens array periodically and increase the spatial sampling density for state unmixing, and a conventional complementary metal–oxide–semiconductor (CMOS) sensor placed at the back focal plane of the microlens array for high-throughput photon detection. **b**, The imaging principle of the meta-imaging sensor. Light from different angles (labelled with different colours) is focused onto different pixels after each microlens. High-resolution 4D spatial–angular measurements are obtained by combining pixels with the same angle based on the microlens positions, which can be used to generate complex-field-modulated images up to the diffraction limit of the imaging lens with ISA. **c**, The prototype camera with the meta-imaging sensor inside, as used in all the experiments. **d**, Optical aberration disturbs the light emitted from the same point, resulting in blurry 2D images or lateral shifts of different angular components. Although all the angular components are mixed coherently in a 2D sensor they are separated in the meta-imaging sensor and can be realigned during post-processing to recover the aberration-corrected high-resolution image; this technique is called digital adaptive optics (DAO). **e**, For spatially nonuniform aberrations, a hardware adaptive optics system with a deformable mirror array can only correct a small FOV, whereas DAO can achieve multisite aberration corrections simultaneously without influencing the data acquisition speed. **f**, Comparisons of the results yielded by a single plastic lens with a 2D sensor, a traditional light-field sensor and the meta-imaging sensor with and without DAO. The same type of CMOS chip was used in each test.

**Extended Data Fig. 1:**
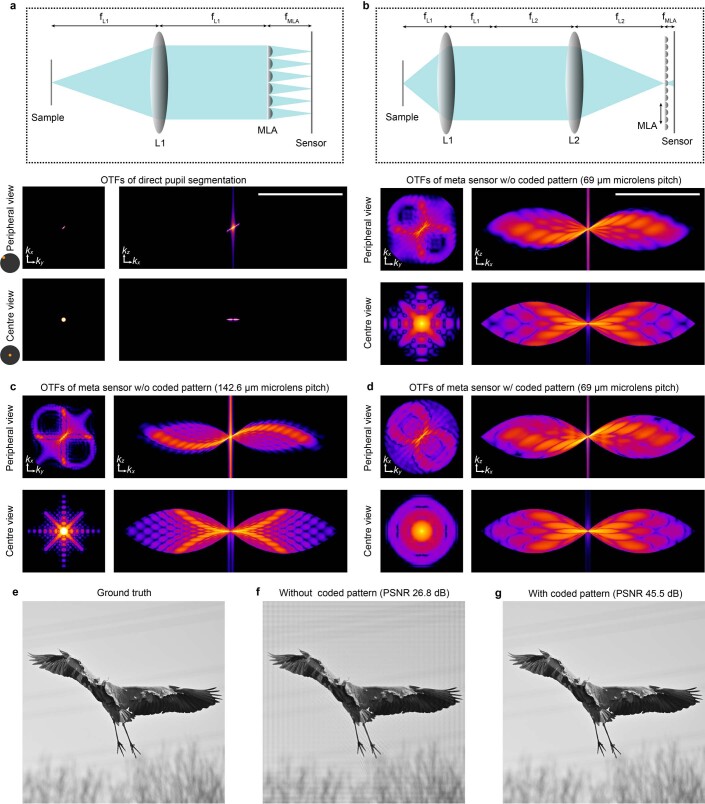
Illustrations of the diffraction effect introduced by the small coded microlens aperture for incoherent synthetic aperture. **a**, The optical transfer functions (OTFs) of different views obtained by direct pupil segmentation, illustrating the loss of spatial resolution. The schematic is shown in the top row, in which the microlens array (MLA) is placed at the pupil plane with a large microlens aperture. **b**, The OTFs of different views obtained by the meta-imaging sensor without the coded pattern. All the simulation imaging parameters are the same as those in the imaging system described in the Methods with the magnification factor of 10 and imaging *f*-number of 1. We set the centre wavelength as 1,000 nm for the convenience of simulation. **c**, The OTFs of different views obtained by the meta-imaging sensor without the coded pattern with a larger microlens aperture and the same *f*-number: 142.6 μm pitch size for 31×31 angles. The nulls in the OTFs increase with a large microlens aperture due to the reduction of the diffraction effect. **d**, The OTFs of different views obtained by the meta-imaging sensor with the circular coded pattern and 69 μm microlens size, illustrating reduced nulls in the OTF. The vertical banding in the OTF at low transverse frequencies is the artefact originated from the fftn function in Matlab due to the large depth of field of the point spread function (PSF). **e**, Ground-truth image used for numerical simulation. **f**, Reconstructed result of the meta-imaging sensor without the circular pattern on each microlens, which exhibits reconstruction artefacts for dense structures. **g**, Reconstructed result of the meta-imaging sensor with the circular pattern on each microlens without artefacts. Scale bar: 1 μm^−1^. PSNR, peak signal-to-noise ratio.

**Extended Data Fig. 2:**
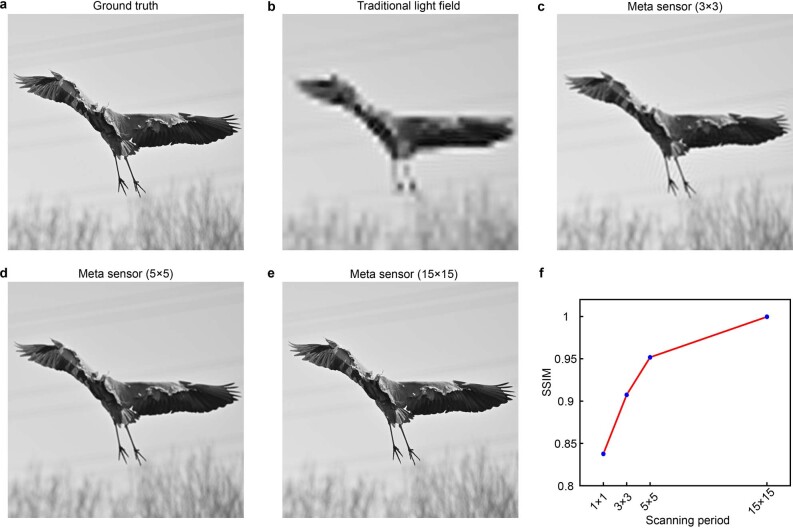
Comparisons of the reconstruction performance with different scanning periods. **a**, Ground-truth image used for numerical simulations. All the imaging parameters are the same as the imaging system described in Methods with a magnification factor of 10 and *f*-number of 1. **b**–**e**, Reconstructed images of the meta-imaging sensor with different scanning periods: 1×1 (**b**), 3×3 (**c**), 5×5 (**d**), and 15×15 (**e**). The scanning period 1×1 corresponds to the traditional light-field camera without the scanning process. **f**, The curve of the structural similarity index measure (SSIM) between the reconstruction and ground truth versus different scanning periods. It can be noted that the reconstruction performance gradually converges with the increase of the scanning number. Therefore, we choose the scanning period of 5×5 for most of the experiments in our manuscript (see Supplementary Table 1).

**Extended Data Fig. 3:**
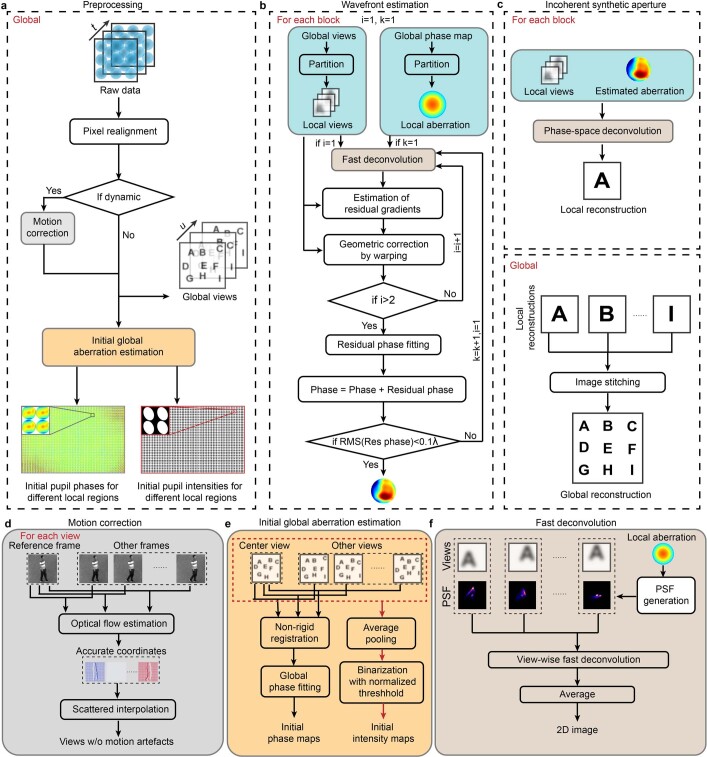
The flow chart of the data processing pipeline for the meta-imaging sensor. **a**, Pre-processing module. The raw data were first converted into different views by pixel realignment. For dynamic samples, motion correction is used to eliminate motion artefacts. Then the global views are segmented into small blocks evenly to estimate and correct the spatially nonuniform aberrations. **b**, Wavefront estimation module for each local block. It can be skipped by using the local system aberration directly to reduce the computational costs. For each local block, we can further estimate the environmental aberrations with an alternating direction method of multipliers (ADMM). We extend the DAO into wave optics by updating the aberrated PSFs with estimated aberrations based on the wave-optics model for each three iterations during the ADMM method. **c**, Incoherent synthetic aperture (ISA). The aberration-corrected image for each local block can be obtained by phase-space deconvolution with the estimated aberrated PSFs. Different blocks are stitched together averaging at the overlapped area for the global reconstruction afterwards. **d**, Motion correction module applied to each view separately. **e**, Initial global aberration estimation used to estimate the spatially nonuniform system aberrations. **f**, Fast deconvolution module. The estimated image during the wavefront estimation step is obtained by the average of the deconvolved results with different angular PSFs based on the hyper-Laplacian prior.

To address these problems, we propose a framework for the meta-imaging sensor by integrating a coded microlens array in a high-speed piezo stage, which is then bonded to a conventional imaging sensor with the photosensitive area located at the back focal plane of the microlens array (Fig. 1a). Each microlens focuses the light from different angles onto different sensor pixels for angular sampling (Fig. 1b, corresponding to 15 × 15 angular pixels). The aperture size of each microlens is only about ten times of the diffraction limit of the image plane, which introduces a diffraction effect to the incoherent light field and preserves the high-frequency information during angle separation through frequency aliasing (Extended Data Fig. 1a,b). By increasing the size of microlens aperture, the diffraction effect will gradually decrease, leading to reduction of spatial resolution (Extended Data Fig. 1c). Different from a previous scanning light-field technique^33^, we further coat a chromium film with a circular pattern on each microlens to block the light passing through the surrounding corners of the square footprint of each microlens (see Methods and Fig. 1a). Such a circular aperture reduces the nulls in the optical transfer functions for artefact-free reconstruction, which is essential for universal imaging scenarios without the sparse prior applied in fluorescence imaging (Extended Data Fig. 1d–g). Then we use high-speed periodic drifting of the microlens array to increase the spatial sampling density limited by the physical size of the microlens, which can unmix the frequency aliasing of ISA (Extended Data Fig. 2a–f). After assembling the pixels with the same angle according to the microlens positions in phase space (Fig. 1b), we apply a deconvolution-based ISA algorithm to obtain a full-resolution focal stack or all-in-focus images with the extended depth of field and diffraction-limited resolution of the imaging lens (see Methods and Extended Data Fig. 3a–f). A high-density depth map can be retrieved simultaneously based on multiview stereo (see Methods). The meta-imaging sensor can directly replace conventional imaging sensors without additional hardware modifications (Fig. 1c).

**Extended Data Fig. 4:**
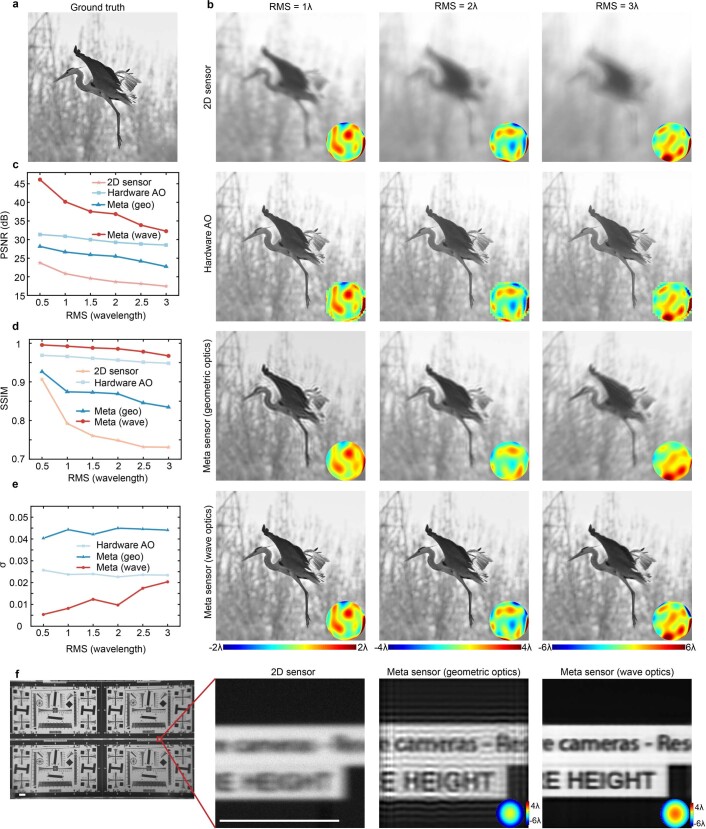
Numerical evaluations of the DAO performance with different aberration levels. **a**, Ground truth of the simulated image. **b**, Results obtained by a conventional 2D sensor, a 2D sensor with hardware adaptive optics (AO) through a tip-tilt-based deformable mirror array (15×15 segments), and the meta-imaging sensor with DAO in geometric optics and wave optics, shown in different rows. Results with different r.m.s. of aberrations (0.5*λ*, 1*λ* and 3*λ*) are shown in different columns. The aberrations are generated randomly with a maximum Zernike order of 45. The simulated imaging settings are the same as aforementioned. The ground truth aberrations are shown in the insets of the 2D sensor results, while the corrected aberrations of different methods are shown in corresponding insets. The colour bars for each column are shown at the bottom. **c**, The curves of peak SNR (PSNR) versus different r.m.s. of aberrations added for different methods. **d**, The curves of SSIM versus different r.m.s. of aberrations added for different methods. **e**, The curves of relative residual wavefront errors *σ* versus different r.m.s. of aberrations added for different methods, where *σ* is calculated through the ratio of the residual phase r.m.s. to the peak-to-valley value of the added aberration. **f**, Results of experimental data obtained by 2D sensor and meta-imaging sensor with DAO in geometric optics and wave optics. Scale bar: 20 mm (**f**). Source data

**Extended Data Fig. 5:**
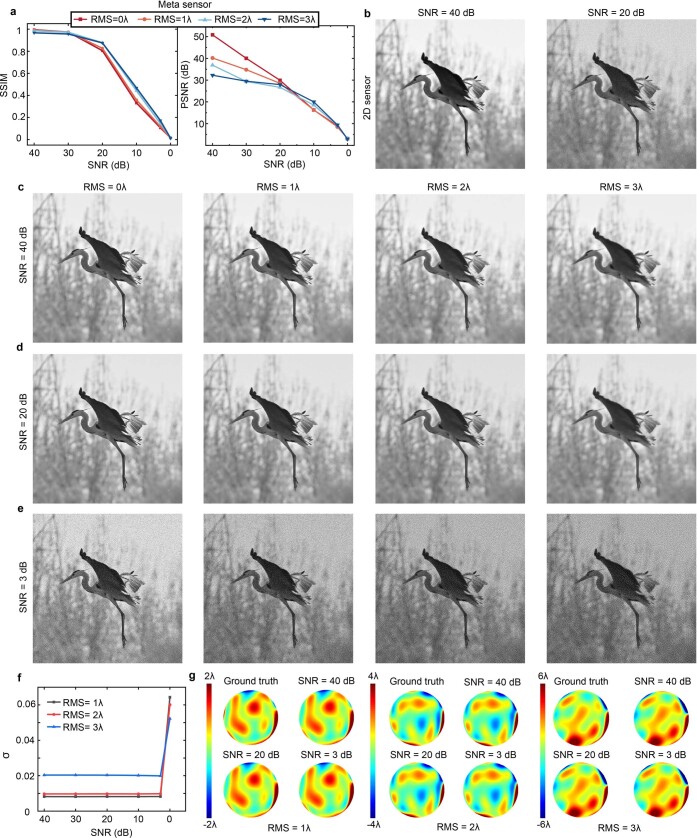
Numerical evaluations of the noise robustness of the meta-imaging sensor. **a**, The curves of the SSIM and peak SNR versus different noise levels indicated by the SNR under different aberration levels (0*λ*, 1*λ*, 2*λ* and 3*λ*). Different levels of Gaussian noises are added to simulate the readout noise. The simulated settings are the same as aforementioned. **b**, Results obtained by a conventional 2D sensor with the SNR of 20 dB (right) and 40 dB (left) without aberrations. **c**–**e**, Results obtained by the meta-imaging sensor with different SNRs: 40 dB (**c**), 20 dB (**d**) and 3 dB (**e**), and aberration levels. **f**, The curves of relative residual wavefront errors *σ* versus different SNRs under different aberration levels (1*λ*, 2*λ* and 3*λ*), showing strong noise robustness of the meta-imaging sensor for aberration estimations. **g**, Ground-truth aberrations and corresponding estimated aberrations with different SNRs. Source data

More importantly, with high-resolution plenoptic light distributions, we can generate complex-field-modulated images precisely in post-processing without additional optical devices, which is the key advantage of the meta-imaging sensor over conventional 2D sensors. A typical example is aberration correction, which is a fundamental problem in optics. Imperfect lenses or turbulence disturb the rays emitted from the same point, leading to severe blurs in 2D sensors. These blurs are difficult to correct without an active device or accurate aberration measurements (Fig. 1d). By contrast, the meta-imaging sensor is more robust to aberrations with reduced crosstalk between angular components, which can be recombined into a perfect focus during post-processing; this is the DAO framework^33^. Here we extend the concept of DAO from geometric optics to wave optics, providing a more accurate description of the imaging model (see Methods). The aberrated phase can be estimated from the relative lateral shifts of the same structure in different views^34^, which can be used to generate accurate 4D PSFs for ISA with aberration corrections. To quantitatively evaluate the performance, we conduct numerical simulations with different aberration levels (Extended Data Fig. 4a,b). Wave-optics-based DAO achieves an approximately 10-dB peak signal-to-noise ratio improvement over geometric-optics-based DAO, with more accurate estimations of aberrated wavefronts. Compared to a hardware adaptive optics approach based on a deformable mirror array with 15 × 15 tip-tilt actuators, DAO obtains similar performance (Extended Data Fig. 4c–e). Experimental results on a resolution test chart further verify the superiority of the wave-optics model (Extended Data Fig. 4f). In addition, the DAO framework exhibits strong noise robustness with consistent aberration estimation performance, because the correlation process utilizes all signals contained in a small region (Extended Data Fig. 5a–g).

By integrating the adaptive optics capability into the sensor, meta-imaging sensor can achieve multisite aberration corrections without reducing the data acquisition speed (Fig. 1e and Supplementary Table 1). Owing to the high nonuniformity of aberration distributions, the effective FOVs of traditional hardware adaptive optics systems are usually very small, leading to a considerable waste of sensor pixels. By contrast, the meta-imaging sensor can apply different aberration corrections for every local region across a wide FOV, which is also hard to obtain with traditional light cameras (Fig. 1f).

## Robust photography with a single lens

The cost and size of camera lenses grow rapidly with the increase of the effective space–bandwidth product, as they usually require multiple well designed lenses with large apertures to correct spatially nonuniform optical aberrations, posing great challenges for lightweight systems or portable devices^6^. Meanwhile, the surface testing of large-aperture optical systems is another challenge faced by many telescopes with respect to aberration characterizations. The meta-imaging sensor provides a scalable distributed solution to these problems from the sensor side.

**Fig. 2:**
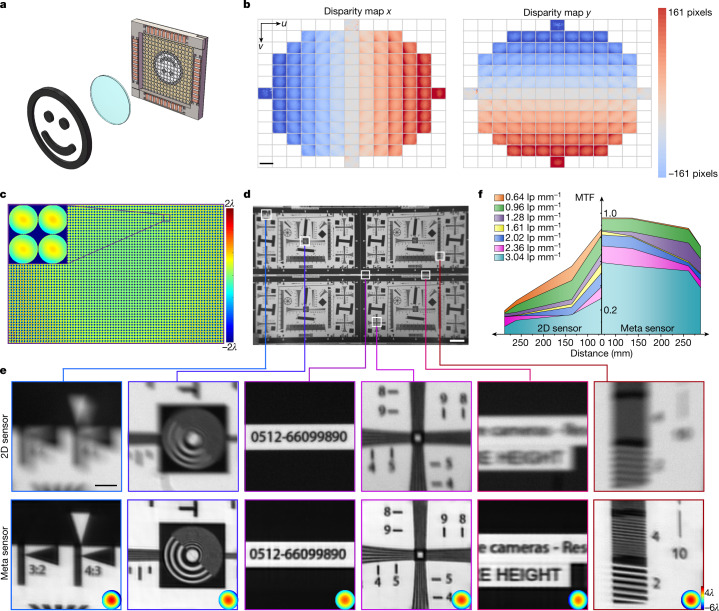
High-performance 48-megapixel imaging with a single 3D-printed plastic lens. **a**, Schematic of the imaging set-up. The plastic lens with a focal length of 135 mm and an effective *f*-number of 10 costs less than 1 US dollar. **b**, Measurements of the spatially nonuniform system aberrations with the meta-imaging sensor by imaging a static sample, using the four resolution charts shown in **d**. The disparity maps for different views (using the centre view as the reference) correspond to the phase gradients at every local region for different subapertures. **c**, Aberrated wavefronts across the whole FOV obtained through the integral of the gradients. Defocus terms are removed for better visualization. **d**, Imaging results of the four resolution charts (ISO 12233). **e**, Magnified regions of **d** show comparisons between the 2D sensor and the meta-imaging sensor, with estimated aberrations shown in the insets. The same type of CMOS chip was used for fair comparisons. **f**, Measured MTFs of the 2D sensor (left) and meta-imaging sensor (right) at different spatial frequencies and different distances from the FOV centre. lp mm^−1^, line pairs per mm. Scale bars: 7,000 pixels (**b**), 40 mm (**d**), 5 mm (**e**). Source data

**Extended Data Fig. 6:**
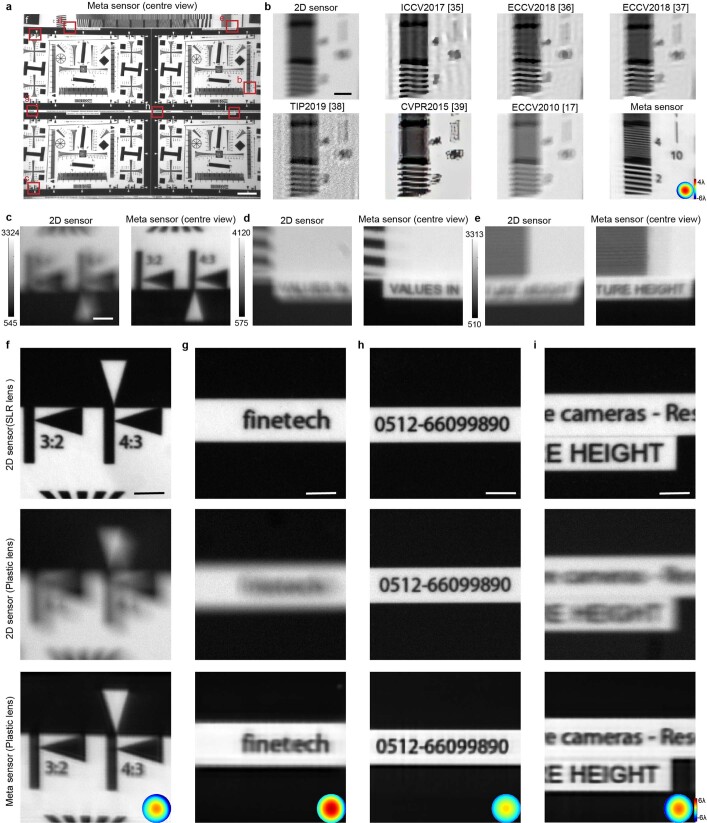
Comparisons between the meta-imaging sensor and the conventional 2D sensor. **a**, The centre-view image obtained by meta-imaging sensor with a single plastic lens. The sample is four resolution charts (ISO 12233), which is the same as Fig. 2d. **b**, Part of the image captured by a conventional 2D sensor with a single plastic lens and corresponding results after applying different blind deconvolution algorithms marked on the top^17, 35–39^. The result obtained by the meta-imaging sensor under the same condition is also shown for comparison with the estimated aberration shown in the inset. **c**–**e**, The magnified regions of the raw data captured by the 2D sensor and meta-imaging sensor under the same imaging conditions and exposure time to show the SNR improvement of the meta-imaging sensor with the existence of strong aberrations. **f**–**i**, Magnified regions marked in **a** obtained by different methods including the 2D sensor with a high-quality single lens reflex (SLR) camera lens (Canon EF70-200mm 1:2.8L), the 2D sensor with a single plastic lens, and the meta-imaging sensor with the same single plastic lens. Estimated aberrations of the local regions are shown in the insets. Scale bars: 40 mm (**a**), 5 mm (**b**–**i**). Source data

**Extended Data Fig. 7:**
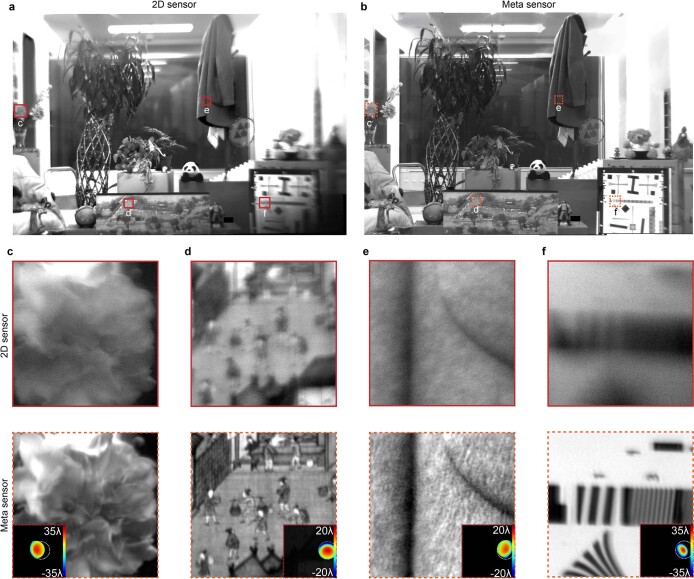
Gigapixel imaging with a single lens by the meta-imaging sensor. **a**, The image obtained by a conventional 2D sensor with a single plano-convex lens. We set up the scenes by ourselves in the lab. A black box is used to block the logo for copyright reasons. A photo of the painting ‘Along the River During the Qingming Festival’ by Qiu Ying was printed as one of the samples to be imaged. **b**, The image obtained by the meta-imaging sensor with a single plano-convex lens. To achieve gigapixel imaging with our current CMOS chip, we fixed the lens and manually shifted the 2D sensor and the meta-imaging sensor in the lateral domain to cover a large field of view (FOV). Then the gigapixel images were obtained by stitching the images with tiny overlaps. The pixel number of both images after stitching was 26,207 × 39,194 (~1.03 gigapixel). A black box is used to block the logo for copyright reasons. **c**–**f**, Magnification of regions marked in **a** obtained by the 2D sensor (top) and the meta-imaging sensor (bottom). The estimated aberrations by the meta-imaging sensor are shown in the inset. The dashed circles represent the telecentric region with a *f*-number of 10.

To demonstrate the superiority of the meta-imaging sensor over 2D sensors, we conducted an experimental comparison by imaging a resolution chart (International Organization for Standardization standard ISO 12233) with a single 3D-printed plastic lens that cost less than 1 US dollar (Fig. 2a). The system aberrations were corrected through a global aberration estimation (see Methods). On the basis of the assumption that the system aberrations change smoothly across the whole FOV, we developed a non-rigid registration algorithm to calculate the disparities between different views and the centre view, corresponding to the gradient maps of aberrated wavefronts (Fig. 2b and Extended Data Fig. 3e). Then, the aberrations at different local regions were obtained through the integration of different subaperture gradients (Fig. 2c). With the multisite DAO approach, the meta-imaging sensor could achieve effective 48-megapixel imaging (limited by the sensor pixel number) with consistent performance across the whole FOV. By contrast, the imaging performance of a conventional 2D sensor with the same type of complementary metal–oxide–semiconductor (CMOS) transistor chip degraded quickly with increasing off-axis distances (Fig. 2d,e). Even with advanced deblurring algorithms, it was difficult to recover the high-frequency information lost by the imaging system (Extended Data Fig. 6a,b)^35–39^. In terms of the MTF, the meta-imaging sensor achieved an approximately fivefold improvement at the edge (Fig. 2f). Moreover, the raw data of the meta-imaging sensor exhibited a much better signal-to-noise ratio than the 2D sensor under the same imaging conditions and exposure time, illustrating the intrinsic aberration robustness of the sensing framework (Extended Data Fig. 6c–e). Although a conventional 2D sensor could achieve similar performance with a high-quality camera lens (Canon EF70-200mm 1:2.8L), the camera lens had a larger size and was 1,000 times more expensive than the 3D-printed plastic lens (Extended Data Fig. 6f–i). With gigapixel CMOS expected to be developed in the near future, the meta-imaging sensor paves the way towards gigapixel imaging in portable devices with compact, low-cost optical systems. We further conducted gigapixel imaging with a single plano-convex lens by shifting the sensors laterally to cover the FOV over 1 gigapixel (Extended Data Fig. 7a–f). The meta-imaging sensor shows better resolution and contrast than the 2D sensor with uniform performance across the wide range.

**Fig. 3:**
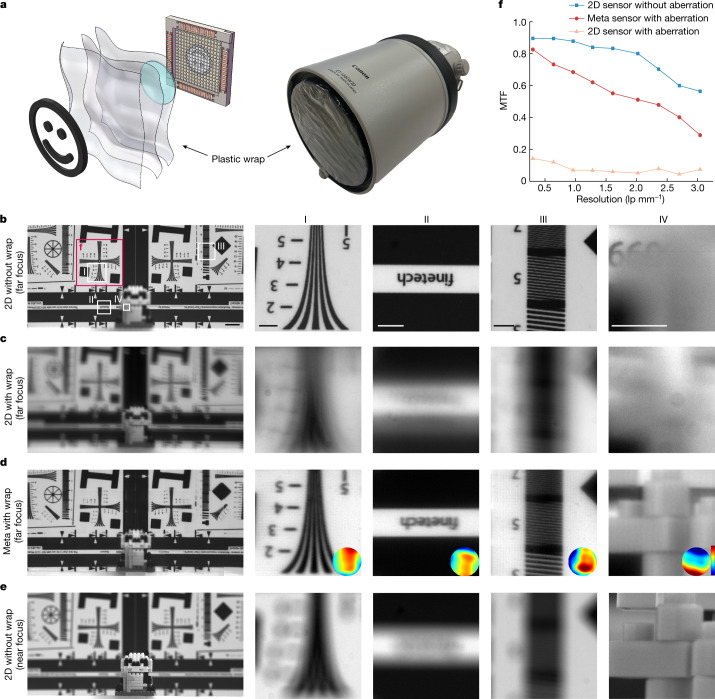
Robust imaging through strong environmental aberrations with an extended depth of field. **a**, Schematic of the experiment in which three pieces of plastic wrap were placed in front of a high-quality camera lens (Canon EF 300mm 1:2.8L) to introduce fixed, nonuniform, strong aberrations. For this scenario, we used the resolution charts to measure the MTF and placed a toy in front of the chart to show the extended depth of field (Supplementary Video 1). **b**–**e**, The regions marked I–IV in **b** are magnified in the right-hand columns. **b**, Image obtained by the 2D sensor when focusing on the resolution chart without the wrap. **c**, Image obtained by the 2D sensor when focusing on the resolution chart with the wrap. **d**, Image obtained by the meta-imaging sensor when focusing on the resolution chart with the wrap. The estimated aberrations are shown in insets, and the colour scale is from 6*λ* (red) to −6*λ* (blue). **e**, Image obtained by the 2D sensor when focusing on the toy without the wrap. **f**, MTF curves calculated based on the region marked in **b** for the 2D sensor with and without the wrap and the meta-imaging sensor with the wrap. Scale bars: 20 mm (**b**–**e**), 5 mm (I–IV). Source data

Additionally, conventional 2D sensors, even equipped with expensive camera lenses, suffer from limited depths of field and environmental aberrations such as water drops and irregular glasses, whereas the meta-imaging sensor is more robust with an extended depth of field. We conducted an experimental comparison by placing three pieces of plastic wrap in front of the lens (Canon EF 300mm 1:2.8L) to introduce environmental aberrations (Fig. 3a and Supplementary Video 1). A toy placed in front of the resolution chart was blurry when the 2D sensor was focused on the resolution chart without the plastic wrap (Fig. 3b). After adding the plastic wrap, we observed severe degradation in the 2D sensor (Fig. 3c), but the meta-imaging sensor could still preserve the resolution with a slight reduction in contrast (Fig. 3b–e). Although stitching artefacts remained across the FOV owing to the partitioning deconvolution and large aberration variances, the performance was still consistent as a result of the multisite DAO capability. The MTF curves show at least a tenfold improvement in resolution (Fig. 3f). Moreover, all the samples were within the extended depth of field of the meta-imaging sensor, which was a dilemma for the 2D sensor (Fig. 3e).

## Optical-flow-based motion correction

**Fig. 4:**
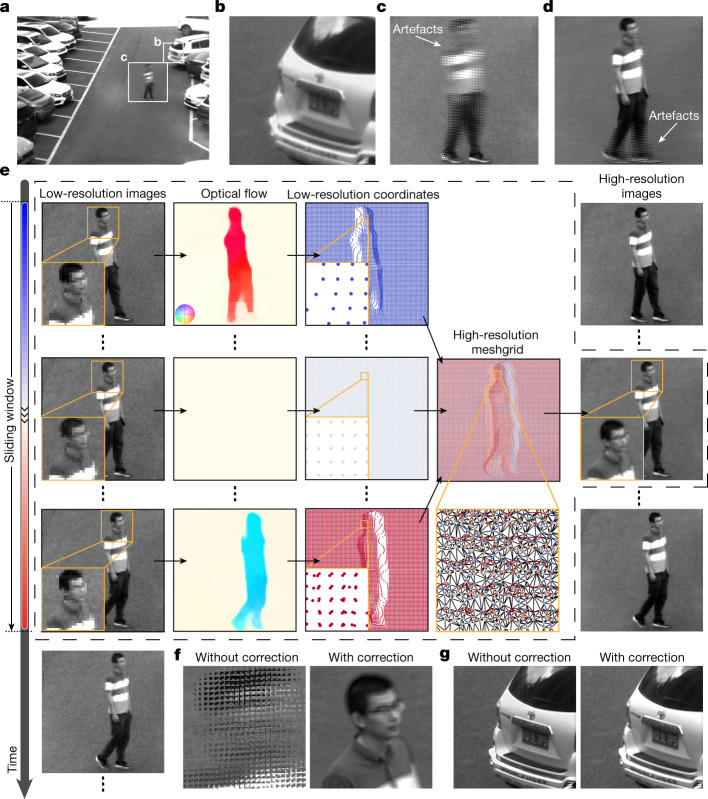
Optical-flow-based motion correction for dynamic scenes. **a**, Central view of the data captured by the meta-imaging sensor without motion correction. The car number plates belonging to others were masked for privacy. **b**, Magnified region marked in **a** for static structures. **c**, Magnified region marked in **a** for dynamic structures, illustrating the artefacts induced by motions during scanning. **d**, Result after rigid motion correction with artefacts remaining owing to nonuniform motions. **e**, Pipeline of the motion-correction algorithm based on optical flow estimations for each view (Supplementary Video 2). A sliding window of the scanning period was used to realign multiple low-resolution images into a high-resolution image at the centre time point with the same temporal sampling rate. The optical flow maps from other low-resolution frames to the centre low-resolution frame were estimated to calculate accurate coordinates for all the low-resolution sampling points in the high-resolution mesh grid at the centre time point (labelled by dots with different colours). Then, the high-resolution image could be obtained through scattered interpolation with dense sampling and accurate equivalent scanning positions for each microlens. The coloured circle in the inset of the optical flow map represents the scale bar of the normalized disparity vectors for the optical flow maps, with different colours corresponding to different disparity vectors in the normalized polar coordinates. **f**, ISA results of the dynamic structures with and without motion correction. **g**, ISA results of the static structures with and without motion correction.

Akin to the motion blur that occurs during exposure with the 2D sensor, motion during scanning results in motion artefacts in the meta-imaging sensor (Fig. 4a–c). Fortunately, most real-world scenes change continuously in the temporal domain; this property can be used to eliminate motion artefacts up to the camera frame rate. However, motions such as human behaviours are highly nonuniform and are difficult for simple rigid registration algorithms to address (Fig. 4d).

Therefore, we developed an optical-flow-based algorithm for time-lapse video to correct nonuniform motion artefacts without reducing the spatial resolution. The whole pipeline was conducted on each view separately (Fig. 4e and Supplementary Video 2). As the microlens array was shifted periodically (5 × 5 frames here), we applied a sliding window (25) to synthesize a high-resolution image from low-resolution images across the scanning period without reducing the temporal sampling density. Specifically, we calculated the optical flow from low-resolution images at different time points to the low-resolution image at the centre time point, and these flow maps were used to generate accurate coordinates of these low-resolution measurements in the high-resolution mesh grid with dense sampling. Then, the high-resolution image without motion artefacts was obtained through the scattered interpolation of 25 low-resolution images with accurate high-resolution coordinates. Finally, high-resolution images of different views were used for ISA without motion artefacts or a speed reduction (Fig. 4f). The proposed algorithm can also effectively preserve the spatial resolution for static scenes (Fig. 4g).

## Turbulence correction for telescopes

Large-scale optical astronomical surveys have produced various important discoveries in astronomy^40^. However, atmospheric turbulence inevitably introduces highly dynamic aberrations, imposing a fundamental limit on the spatial resolution of ground-based telescopes^41^. Hardware adaptive optics techniques can alleviate this problem and facilitate broad applications, although it involves high expenses and suffers from small effective FOV (usually less than 40 arcseconds in diameter), owing to nonuniform aberration distributions^5^. The meta-imaging sensor provides a great opportunity for large-aperture survey telescopes with multisite DAO capability by simply replacing the imaging sensor.

**Fig. 5:**
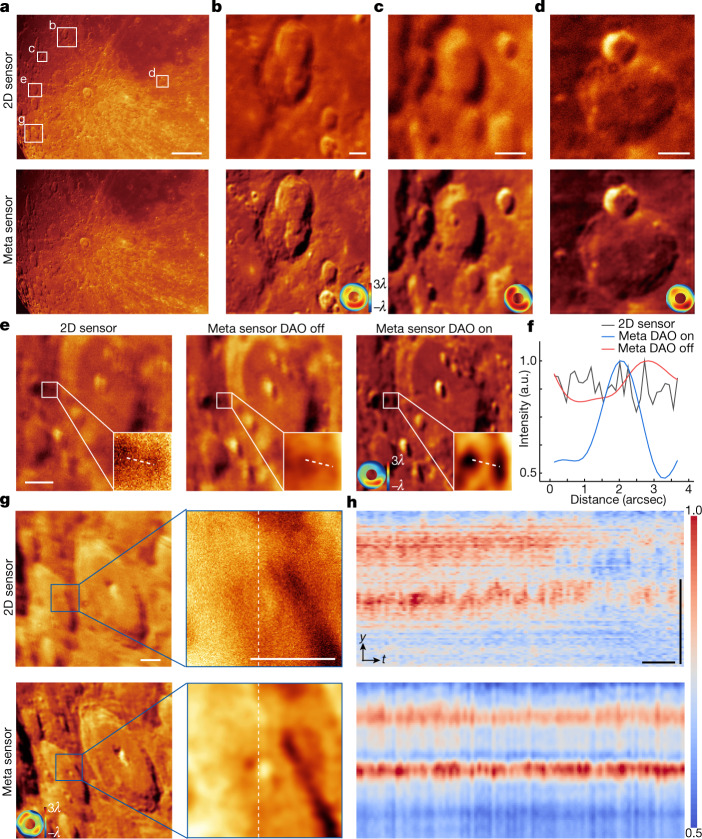
Multisite DAO against dynamic turbulence for ground-based telescopes. **a**, Images obtained by the 2D sensor and meta-imaging sensor with the Tsinghua-NAOC 80-cm telescope at 21:12 (GMT+8) on 25 March 2021 (Supplementary Video 3). **b**–**d**, Magnified regions marked in **a** with estimated aberrations shown in the insets. The pupil functions are ring-shaped. **e**, Comparisons of the regions marked in **a** obtained by the 2D sensor and the meta-imaging sensor with and without DAO, illustrating the effectiveness of DAO. **f**, Cross-sectional profiles of the dashed lines marked in **e**. **g**, Magnified regions of the Morey Crater marked in **a** obtained by the 2D sensor and the meta-imaging sensor. **h**, Kymographs of dashed lines marked in **g** for about 10 s. Scale bars: 100 arcseconds (**a**), 10 arcseconds (**b**–**g**), 10 arcseconds vertically, 1 s horizontally (**h**). Source data

**Extended Data Fig. 8:**
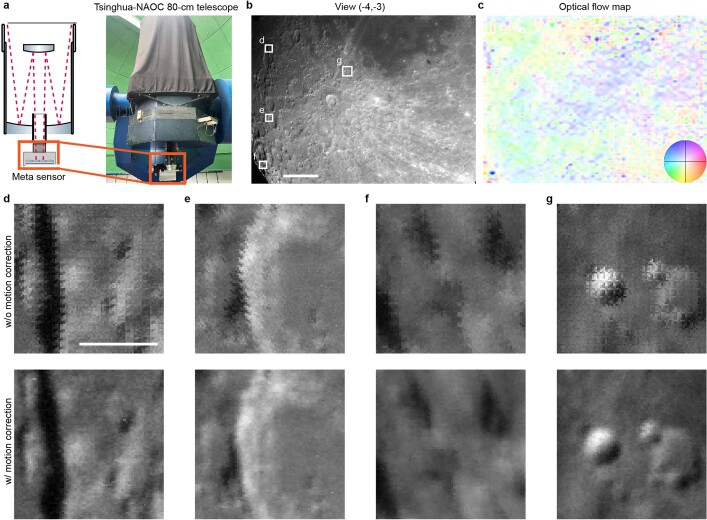
Motion correction for dynamic turbulence on an 80-cm ground-based telescope. **a**, We directly placed our meta-imaging sensor or the conventional 2D sensor containing the same CMOS chip on the image plane of the Tsinghua-NAOC 80-cm telescope. The telescope is an 80-cm Cassegrain telescope. The photograph showing our proof-of-concept camera attached on the telescope during experiments. **b**, The view (−4, −3) after direct pixel realignment of the Moon imaged by the meta-imaging sensor without motion correction. **c**, The optical flow map from the adjacent time point to the central time point obtained by the non-rigid registration algorithm. **d**–**g**, Magnification of regions marked in **b** to better visualize the view data without (top) and with (bottom) motion corrections, illustrating the effectiveness of the algorithm. Scale bars: 100 arcseconds (**b**,**c**), 10 arcseconds (**d**–**g**).

To verify its effectiveness, we compared our meta-imaging sensor with a conventional 2D sensor containing the same CMOS chip on the Tsinghua-NAOC 80-cm telescope at the Xinglong Observatory of the National Astronomical Observatories of China (NAOC) (Extended Data Fig. 8a). We chose the Moon as the target. Similar to sample motions, the low-resolution images changed quickly during scanning owing to dynamic aberrations caused by turbulence, and the aberration distribution remained smooth across the whole FOV. Therefore, we used the non-rigid registration algorithm here to estimate the smooth optical flow map and compensate for the motion artefacts (Extended Data Fig. 8b–g). Then, the aberration-corrected all-in-focus image was obtained through ISA with multisite DAO (Fig. 5a–d). The meta-imaging sensor achieved much better resolution and contrast than the conventional 2D sensor without other hardware modifications across the whole FOV (covering over 1,000 arcseconds in diameter). A larger FOV could be directly obtained with a larger sensor. Although the meta-imaging sensor itself was robust to aberrations, wave-optics-based DAO was necessary to resolve minute structures, which could not be distinguished with 2D sensors (Fig. 5e,f). Moreover, we found that the meta-imaging sensor achieved consistent robust performance over a long term, but the results of the 2D sensor distorted quickly with turbulence (Fig. 5g,h). The improved resolution obtained by the meta-imaging sensor is hard to achieve by selecting out the clearest image from a time-lapse video captured with a 2D sensor (Supplementary Video 3). Moreover, better resolution can be obtained in telescopes with larger apertures via the meta-imaging sensor, whereas the resolution of 2D sensors has reached the boundary set by turbulence.

## 3D imaging and industrial inspection

**Fig. 6:**
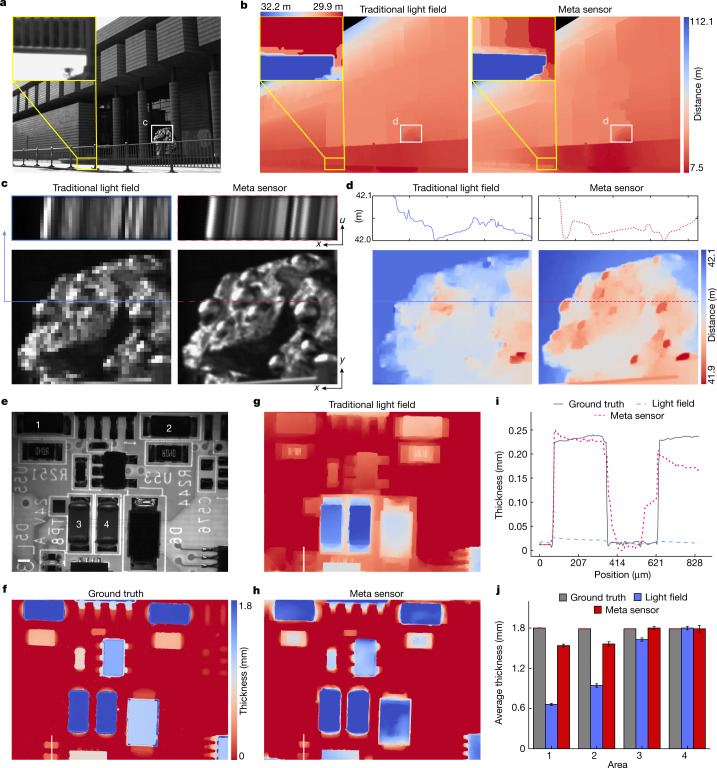
High-speed megapixel depth sensing for autonomous driving and industrial inspection. **a**, Image of the Tsinghua University Art Museum captured by the meta-imaging sensor. **b**, Reconstructed depth maps of the traditional light-field sensor (without scanning) and meta-imaging sensor for comparisons with the magnified regions shown in the insets. **c**, Magnified regions of the raw data obtained by the traditional light-field camera (without scanning) and the meta-imaging sensor. Both the centre view and the epipolar plane images of the marked lines are shown. **d**, Magnified depth maps of the same region as in **c**, for comparisons with the depth profiles of the marked lines. **e**, Image of a circuit board obtained by the meta-imaging sensor with a 0.15 NA (numerical aperture) objective. **f**, Ground truth of the depth map captured via commercial focus-variation microscopy for half an hour. **g**, Depth map obtained by the traditional light-field camera after system aberration correction. **h**, Depth map obtained by the meta-imaging sensor after system aberration correction. **i**, Depth profiles of the white lines marked in **f**–**h**. **j**, Average thicknesses of the four components of the same type obtained by different methods. The centre values represent the average. Error bars represent 1 standard deviation (*n* = 14,400 pixels for each component). Scale bar: 1 mm (**e**–**h**). Source data

**Extended Data Fig. 9:**
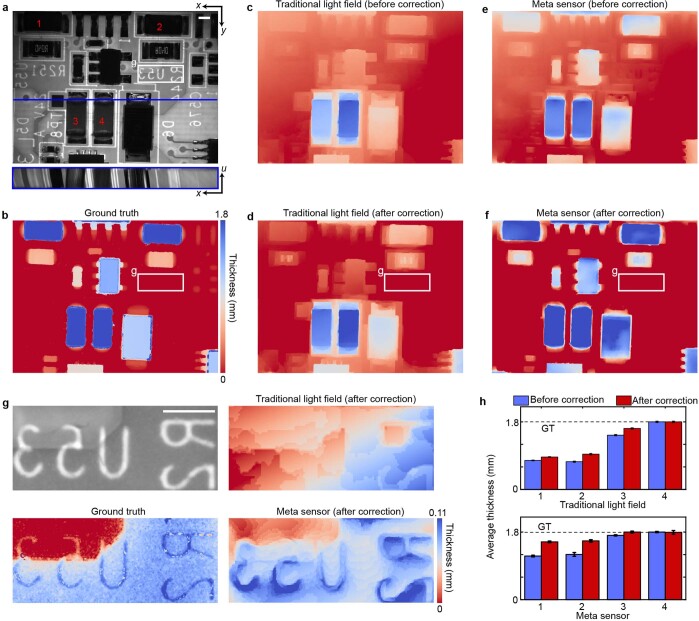
Aberration correction for accurate depth sensing with the meta-imaging sensor. **a**, Central view of a circuit board obtained by our meta-imaging sensor. The spatial–angular plane of the blue solid line is shown in the bottom. Different depths can be estimated from the slopes of the corresponding points. **b**, The ground-truth depth map obtained by a commercial focus-variation microscopy. **c**,**d**, The depth maps obtained by traditional light-field camera (corresponding to the meta-imaging sensor without scanning) without (**c**) and with (**d**) aberration correction. **e**, **f**, The depth maps obtained by the meta-imaging sensor without (**e**) and with (**f**) aberration correction. **g**, Magnification of the region marked in **a** to show the difference of minute structures. **h**, The average thickness of four components marked in **a** for both traditional light field and meta-imaging sensor with and without aberration corrections. The centre values represent the average. Error bars represent 1 standard deviation (*n* = 14,400 pixels for each component). Scale bars: 1 mm.

It is worth noting that besides wide-FOV high-resolution imaging in a complex environment, depth information can also be retrieved simultaneously via meta-imaging sensors, with higher precision in both the lateral and axial domains than for traditional light-field cameras, providing a low-cost solution for autonomous driving (Fig. 6a,b). As shown in Fig. 6c and Extended Data Fig. 9a, the samples located at different depths have different slopes in the spatial–angular domain; these slopes can be used to infer depths with existing estimation algorithms^42^. The higher spatial sampling density not only increases the lateral resolution but also increases the depth accuracy (Fig. 6d and Supplementary Video 4). Otherwise, it is difficult to distinguish different depths when the maximum lateral shifts of the corresponding pixel at different views are smaller than the sampling interval.

We further demonstrated the application of the meta-imaging sensor in industrial inspections, which require both high-throughput imaging and 3D sensing (Supplementary Video 5). A circuit board was imaged by the meta-imaging sensor with and without scanning (corresponding to the traditional light-field camera) under a 0.15-NA (numerical aperture) objective within 1 s (Fig. 6e). Commercial focus-variation microscopy^43^ was used to obtain ground-truth measurements, which took approximately half an hour to cover the whole depth range (Fig. 6f and Extended Data Fig. 9b). Before depth estimation, we applied the global aberration correction in geometric optics to correct system aberrations (Extended Data Fig. 9c–f). By applying the same light-field depth-estimation algorithm with optimized parameters, the meta-imaging sensor achieved higher resolution and a more precise depth map than traditional light-field cameras (Fig. 6g,h). The meta-imaging sensor could even retrieve minute structures that were hard to distinguish with traditional light-field images (Fig. 6i and Extended Data Fig. 9g). By analysing the heights of four components of the same type, we found that the meta-imaging sensor reduced the depth error across the large FOV (Fig. 6j and Extended Data Fig. 9h).

## Discussion

There are two critical characteristics for ISA with DAO in the meta-imaging sensor. One is the necessity of the wave-optics model for high-resolution reconstruction with aberration correction. The optical aberrations will not only shift the angular PSFs but also slightly change their intensity distributions, which has not been considered in previous DAO frameworks^33^. Therefore, we used phase-generated PSFs during iterations in wave-optics DAO, leading to better image synthesis and more accurate aberration estimations (Extended Data Fig. 4c–e). The other point concerns the integrated scanning process of the microlens array, providing virtual spatial overlap between adjacent microlenses that unmixes the high-frequency information for ISA up to the diffraction limit of the imaging lens^44,45^. Such a scanning process addresses the intrinsic trade-off between spatial and angular resolutions, whereas high spatial resolution in traditional light field, either with focused or unfocused schemes, comes at the cost of reduced angular resolution and depth of field^46^.

**Extended Data Fig. 10:**
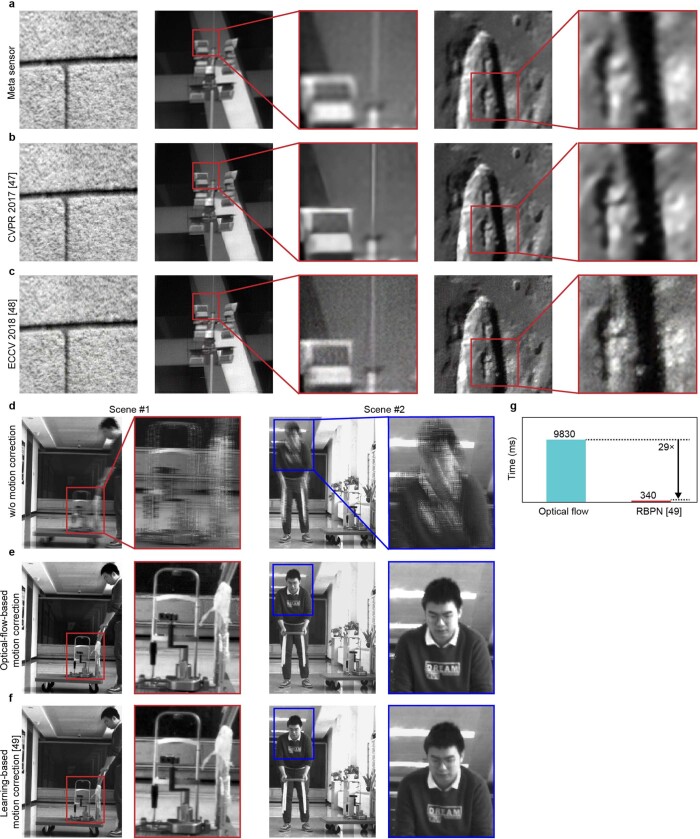
Deep-learning-based methods applied to the meta-imaging sensor. **a**, The imaging results obtained by the meta-imaging sensor with magnifications for better visualization. **b**,**c**, The corresponding results after super-resolution by two different deep-learning-based methods^47,48^ with pre-trained models to further improve the resolution with the data prior, indicating that the meta-imaging sensor is compatible with existing signal processing algorithms. **d**, The central views of the dynamic scenes obtained by the meta-imaging sensor without motion correction. **e**, The corresponding results after our optical-flow-based motion correction. **f**, The corresponding results after learning-based motion correction with a pretrained deep neutral network, a recurrent back-projection network (RBPN)^49^. **g**, Comparisons of the processing time required for motion correction of a single view with 400 × 400 pixels, illustrating about 29-times acceleration achieved by the learning-based method.

Similar to a conventional imaging sensor, learning-based methods can be adopted with pre-trained models to further improve the output of the meta-imaging sensor using data priors (Extended Data Fig. 10a–c)^47,48^. Moreover, given specific applications, more advanced algorithms with parallel computing devices can be designed to achieve either better performance or reduced computational costs. Taking, for example, high-performance photography with a single lens, the system aberrations can be calibrated in advance once only, without the requirement of additional aberration estimations in use. Deep neural networks can be used to accelerate every step of the reconstruction process for real-time applications. As shown in Extended Data Fig. 10d–g, we used a pre-trained recurrent back-projection network^49^ to conduct motion correction for dynamic objects during scanning. Although we can observe slight resolution reduction in learning-based results, the processing time can be reduced by 29 times on a desktop computer. Besides, whereas the current meta-imaging sensor is designed for greyscale images, a multicolour meta-imaging sensor can be developed by utilizing angular redundancy for spectrum coding without reducing the spatial resolution^50^. Chromatic aberrations can be modelled during ISA. The meta-imaging sensor is also compatible for low-light applications such as fluorescence microscopy^33^. Note that hardware adaptive optics and DAO are not in conflict; instead, they can be combined together for more robust aberration correction. By integrating the flexibility of precise optical modulations for incoherent light digitally, we believe the proposed meta-imaging sensor opens new horizons for computational imaging in practical, universal applications with orders-of-magnitude superiority, inaccessible for traditional 2D sensors.

## Methods

### Experimental set-up

The meta-imaging sensor was built upon an existing CMOS sensor with a vibrating coded microlens array (MLA) bonding in front of the photosensitive area, as shown in Fig. 1a. For the proof-of-concept system, we chose a 48-megapixel CMOS sensor (CMOSIS CMV50000, 7,920 × 6,004 pixels) with the Flare 48M30-CX camera (IO Industry) for high-throughput detection. The sensor has a pixel size of 4.6 μm with a maximum frame rate of 30 Hz for whole FOV. The pitch size of the MLA is 69 μm to match the size of 15 × 15 sensor pixels, corresponding to 225 angular measurements. Because most lenses used for experiments have a *f*-number around 10, we chose the focal length of the MLA as 690 μm, leading to the diffraction limit of 5 μm for the wavelength of 500 nm at the back focal plane of the MLA. Smaller focal length of the MLA can also be selected for the camera lens with a large numerical aperture (NA) at the cost of depth of field.

Compared with the diffraction limit of the imaging lens, the aperture size of each microlens should be small enough to introduce the diffraction effects at the image plane for frequency aliasing, which can avoid the resolution degradation due to pupil segmentation for angular samplings (Extended Data Fig. 1). Different from the previous scanning light-field method with a rectangular aperture for each microlens^33^, we coated a chromium film with a circular pattern on each square microlens to ensure light only passes through each microlens via a circular aperture, instead of the surrounding corners of the square footprint of the microlens. The diameter of the circular aperture is also 69 μm. The circular aperture on each microlens can then reduce the nulls in the optical transfer functions for different views, which is essential for artefact-free reconstruction of universal imaging applications without the sparse prior applied in fluorescence imaging (Extended Data Fig. 1d). We then compared the results with and without circular patterns in numerical simulations, demonstrating the effectiveness of the coded diffractive pattern to eliminate the reconstruction artifacts for dense structures (Extended Data Fig. 1e–g).

The coded MLA is then fixed on a piezo stage (P16XY100, CoreMorrow), which facilitates accurate periodic scanning to increase the spatial sampling density of the spatial–angular measurements at high speed. The scanning process can cover all the spatial information and create virtual overlaps between adjacent microlenses to address the frequency aliasing problem for ISA, akin to the ptychographic process^44,45^. As the photosensitive area of the CMOS chip should be placed at the back focal plane of MLA to maximize the depth of field^46^, we removed all the glasses above the chip. To accurately fix the MLA about 690 μm in front of the photosensitive area, we used a linear translation stage (DHC, GCM-V25M) to fine-tune the piezo stage in the axial domain and determined the optimal position when the images after adjacent microlenses were tangent using a camera lens with an *f*-number of 10. A compact five-axis stage (PY005, Thorlabs) was used to slightly adjust the pitch and yaw of the MLA during alignment, so that the MLA could be parallel to the CMOS chip for uniform performance across a large FOV. Finally, all these devices were tightly packaged together with good heat dissipation to be used as a single meta-imaging sensor.

There are four different scanning modes of our meta-imaging sensor, created by shifting the MLA with different periods: 1 × 1, 3 × 3, 5 × 5 and 15 × 15. Longer periods correspond to smaller shifting intervals with a higher spatial sampling density. The maximum displacement of the MLA is smaller than the pitch size of a microlens. We used 1 × 1 scanning to capture the measurements of traditional light-field cameras for comparisons. Numerical simulations on dense structures were conducted to show the influence of different scanning periods (Extended Data Fig. 2). We find that the reconstruction performance reaches convergence with a period of 5 × 5 in terms of structural similarity index measure (SSIM). Therefore, we used 5 × 5 in most of our experiments to reduce motion artefacts during scanning.

The hardware synchronization of the meta-imaging sensor was divided into three stages and obtained with a multifunction I/O device (USB-6363, National Instruments). First, we triggered the piezo stage to shift the MLA to the next position. Then, we set a period of delay before triggering the camera exposure, which usually lasted for 5 ms to wait for the movement of the piezo. Finally, the CMOS chip was triggered for exposure. The readout time of the CMOS chip can be overlapped with the delay period.

For all the experiments, the meta-imaging sensor was directly placed at the image plane of existing optical systems. We used the same type of CMOS chips for fair comparisons between the meta-imaging sensor and traditional 2D sensors placed at the same position. For ISA with multisite DAO, the raw images obtained by meta-imaging sensor went through the same data-processing pipeline including pre-processing (pixel realignment, motion correction and initial global aberration estimations), wavefront estimations for different local regions, and incoherent synthetic aperture (Extended Data Fig. 3). It is noted that initial global aberration estimation can be conducted only once for a specific imaging system. Detailed imaging conditions for all the experiments, including lens type, lens specifications, exposure time, frame rate, scanning period and block sizes for multisite DAO are illustrated in Supplementary Table 1.

### Pixel realignment

Pixel realignment is the main pre-processing step to convert the raw measurements of the meta-imaging sensor *L*(*x*, *y*, *t*) into high-resolution spatial–angular measurements *V*_h_(*x*, *y*, *u*, *v*, *t*). As shown in Fig. 1b, different sensor pixels after each microlens correspond to different angles of the light or different views with coordinates of (*u*, *v*), with the central view located at (0, 0). Each microlens samples a local spatial region with a coordinate of (*x*, *y*), corresponding to the centre position of the microlens. Owing to the physical size limit, the minimum sampling interval of a single light-field image is the microlens diameter. Scanning of the microlens can increase the sampling density and create virtual overlap between adjacent microlenses. Then we assemble the sensor pixels of the same view (*u*, *v*) within a scanning period together based on the centre position of its corresponding microlens (*x*, *y*) to obtain high-resolution spatial–angular measurements *V*_h_(*x*, *y*, *u*, *v*, *t*) with the sampling interval five times smaller than the microlens diameter for the scanning period of 5 × 5, which is called pixel realignment. The sliding window in the temporal domain with a window size of the scanning period can preserve the temporal sampling density of the meta-imaging sensor. Before realignment, the raw light-field image was also resized and rotated to guarantee that each microlens covers about 15 × 15 sensor pixels. Such a rectification process is necessary to calibrate the system error caused by the alignment and microlens fabrications, which increases the robustness of the framework. It takes approximately 0.95 s for 5 × 5 scanning light-field images with a pixel number of 8,000 × 6,000 to conduct rectification and pixel realignment on a desktop computer with a graphical processing unit (CPU, Intel i9-10940X; RAM, 128 GB; GPU, NVIDIA GeForce RTX Titan).

### Motion correction

For highly dynamic samples moving during the scanning process of the meta-imaging sensor, motion artefacts arise after pixel realignment. In previous scanning light-field techniques, a time-weighted algorithm was developed to eliminate the motion artefacts at the cost of spatial resolution degradation^33^. Here we propose an optical-flow-based algorithm for motion correction without reducing the spatiotemporal resolution by exploiting the spatiotemporal continuity in nature. The whole pipeline and pseudocode are shown in Fig. 4e and Extended Data Fig. 3d. We find that motion artefacts originate from incorrect pixel realignment of the pixels from different light-field images captured at different time points, because the actual spatial sampling positions of these microlenses are shifted with the sample movements. Fortunately, most of these shifts still fall in the wide FOV of the meta-imaging sensor, because objects usually move continuously in the scene. Otherwise, it is also hard for conventional imaging sensors to capture the dynamics owing to the motion blur. As long as we can estimate the sample motions accurately, we can still retrieve dense measurements from adjacent frames with accurate spatial sampling coordinates. Therefore, we conduct the motion correction algorithm on each view separately during pixel realignment based on the optical-flow maps. For the motions induced by samples, we use the same optical-flow map estimated from the central view (or the view with the highest sharpness for the conditions without a central view) for all the views. For motions induced by dynamic aberrations, the optical flow maps of different views should be estimated separately. Taking the scanning mode of 5 × 5 as an example, we apply a sliding window to synthesize the high-resolution image *V*_h_(*x*, *y*, *u*, *v*, *t*) at the time point *t* from adjacent low-resolution images *V*_l_(*x*, *y*, *u*, *v*, *t* + *k* − (*T* + 1)/2) (*k* = 1, 2, 3, …, *T*) (with a low spatial sampling density at the interval of the microlens diameter) across the scanning period *T* = 25 without reducing the temporal sampling density. To obtain the accurate coordinates of all the measurements at the time *t*, we apply a state-of-the-art optical flow algorithm^51^ to calculate the flow maps (∆*x*, ∆*y*) from all the low-resolution images *V*_l_(*x*, *y*, *u*, *v*, *t* + *k* − (*T* + 1)/2) to the low-resolution image *V*_l_(*x*, *y*, *u*, *v*, *t*) at the time point *t*. Then the corresponding sampling coordinates of the low-resolution measurements in the high-resolution mesh grid can be represented as:

$$({x}^{{\prime} },{y}^{{\prime} })=(x+\Delta x,y+\Delta y)$$

Finally, the high-resolution image *V*_h_(*x*′, *y*′, *u*, *v*, *t*) without motion artefacts can be obtained through a scattered interpolation method^52,53^ of these 25 low-resolution images based on the accurate dense sampling coordinates.

For turbulence-induced motions, we use a non-rigid registration algorithm to estimate the optical flows, owing to its smooth distributions, which is the same as the method for system aberration estimations. It takes approximately 45 s for 5 × 5 scanning light-field images with a pixel number of 1,000 × 1,000 to conduct motion correction with pixel realignment for all the views on a desktop computer with a graphical processing unit (CP, Intel i9-10940X; RAM, 128 GB; GPU, NVIDIA GeForce RTX Titan). The results of the dynamic samples validate the effectiveness of the algorithm (Fig. 4f and Extended Data Fig. 8). The results of the static samples indicate that the spatial resolution can be preserved very well (Fig. 4g).

### Initial global aberration estimation

Because the spatially nonuniform system aberrations of the existing optical systems are fixed, we first usually conduct an initial global aberration estimation for a specific imaging system (Extended Data Fig. 3e). It is not essential for the meta-imaging sensor. However, it can greatly reduce the computational costs for practical applications without the requirement to correct dynamic environmental aberrations, because the system aberrations can be calibrated only once in advance by imaging any scenes with enough textures. For single-lens imaging experiments and industrial inspection experiments, we used a checkboard with a block size of about 200 × 200 sensor pixels to estimate the global nonuniform system aberrations. The system aberration *A*(*u*, *v*, *x*, *y*)exp[j*φ*(*u*, *v*, *x*, *y*)] with coordinates (*u*, *v*) at the pupil plane can be divided into two parts including the pupil phase distributions *φ*(*u*, *v*, *x*, *y*) and pupil intensity distributions *A*(*u*, *v*, *x*, *y*), owing to the non-telecentricity of common imaging systems. Here j is the imaginary unit. For the aberration wavefronts *φ*(*u*, *v*, *x*, *y*) at different local regions (*x*, *y*) of the image plane, the local phase gradients at different subaperture region (d*φ*/d*u*, d*φ*/d*v*) = *c*(Δ*s*_*x*_, Δ*s*_*y*_) can be obtained through the disparities (Δ*s*_*x*_(*u*, *v*, *x*, *y*), Δ*s*_*y*_(*u*, *v*, *x*, *y*)) from different views *V*_h_(*x*, *y*, *u*, *v*) to the central view *V*_h_(*x*, *y*, 0, 0) with a constant *c* related to the system magnification and sensor pixel size (Fig. 2b). On the basis of the assumption that the system aberrations change smoothly across the whole FOV, the disparities are estimated with a non-rigid registration algorithm. Then the aberration wavefronts can be obtained through an integral as below:

$$\phi \left(u,v,x,y\right)=c\iint {(\Delta {s}_{x},\Delta {s}_{y})}_{({V}_{{\rm{h}}}(x,y,u,v)\to {V}_{{\rm{h}}}(x,y,0,0))}{\rm{d}}u{\rm{d}}v.$$

The non-rigid registration algorithm is implemented by solving the following optimization problem in Pytorch1.9.0 with the Adam optimizer:

$$\mathop{\min }\limits_{\Delta {s}_{x},\Delta {s}_{y}}{\left\Vert {V}_{{\rm{h}}}(x+\Delta {s}_{x},y+\Delta {s}_{y},u,v,t)-{V}_{{\rm{h}}}(x,y,0,0,t)\right\Vert }_{2}^{2}.$$

Where ||•||_2_ corresponds to the L2 norm. For each view during optimization, we manually set 20 × 15 × 2 control points across the whole FOV to fit the shift maps with a smooth distribution.

For the aberration intensity *A*(*u*, *v*, *x*, *y*) at different local regions (*x*, *y*), we can infer the pupil shape from the angular intensity distributions. In practice, we average the intensities of different views within a fixed block for multisite DAO (see Supplementary Table 1). Then the aberration pupil shapes at different local regions are obtained by binarization of the angular intensity distributions with the normalized threshold (similar performance can be obtained for the threshold ranging from 0.5 to 0.8). Such a process is essential for the ISA of the meta-imaging sensor in universal imaging applications, because most regions of most imaging systems are not telecentric, with the chief rays not perpendicular to the image plane. Another example is the Cassegrain telescope with a ring structure for pupil intensity modulations. All this information can be estimated directly from the precise high-dimensional measurements of the light field by the meta-imaging sensor.

Once we obtain the global nonuniform aberration distributions, they can be used as initial local aberrations for further wavefront estimations caused by environmental dynamics such as turbulence, or directly for ISA with aberration correction in conditions without the requirement to correct environmental aberrations. It takes approximately 450 s for 5 × 5 scanning light-field images with a pixel number of 8,000 × 6,000 to conduct global system aberration estimation on a desktop computer (CPU, Intel i9-10940X; RAM, 128 GB; GPU, NVIDIA GeForce RTX Titan).

### ISA with multisite DAO in wave optics

For incoherent light, we can only detect the intensity distributions with the phase information lost due to the temporal average. Therefore, when we directly segment the pupil plane into different subapertures to sample the angular intensity distributions, the spatial resolution is intrinsically reduced owing to the loss of high-frequency information (Extended Data Fig. 1a). Fortunately, we find that MLA at the image plane with a small size of each microlens can address this intrinsic trade-off between spatial and angular resolutions. The circular aperture on each microlens with a size about ten times of the diffraction limit at the image plane adds additional coherence to the incoherent light field, preserving the high-frequency information in the low-frequency region of the optical transfer functions for different views, akin to the structured illumination microscopy^54^ (Extended Data Fig. 1). The scanning process further increases the spatial sampling density to address the frequency aliasing problem and unmix the coded high-frequency information. Then, such additional coherence can be reflected by the variances of different angular PSFs with different emphasis in the spatial frequency domain, which can be used with phase-space deconvolution for ISA^55^ (Extended Data Fig. 3c). Moreover, we find that the periodic circular diffractive patterns can eliminate the reconstruction artefacts for universal imaging applications with dense structures by reducing the nulls in the optical transfer functions (Extended Data Fig. 1d–g).

As shown in Fig. 1d, optical aberrations distort the light emitted from the same point into different positions on the image plane, leading to degraded spatial resolution and contrast for traditional 2D sensors. By capturing the high-resolution spatial–angular information, the meta-imaging sensor reduces the coherent crosstalk between different views and keeps the photons focused within the extended depth of field, facilitating better aberration robustness than traditional 2D sensors, which is important for aberration estimation and correction in post-processing during ISA (Extended Data Fig. 6c–e). Because the optical aberrations are usually nonuniform across a large FOV, we can segment the whole FOV into small blocks equally with 20 pixels overlap (detailed block sizes are shown in Supplementary Table 1). We assume the aberration is uniform in each block. Then we can conduct multisite aberration estimation and correction in post-processing without influencing the data acquisition speed. Finally, high-resolution images after ISA with multisite DAO are stitched together (Extended Data Fig. 3c).

For each local region, the DAO process is divided into two parts: wavefront estimation and ISA. When we do not need to correct the environmental aberrations or other additional aberrations, the wavefront estimation can be skipped by using the local system aberration obtained through initial global aberration estimation directly to reduce the computational costs. For further estimation of the environmental aberration, we apply an alternating direction method of multipliers (ADMM) method^56^ to update the aberrated wavefronts *φ*(*u*, *v*) iteratively as below:

$$\{\begin{array}{c}{H}_{i}(x,y,u,v)=PSFGen(A(u,v){e}^{j{\phi }_{i}(u,v)})\\ {I}_{i}(x,y)=\frac{1}{{N}_{angle}}\sum _{u,v}(Deconv({V}_{h}(x+\Delta {s}_{x}^{(i)}(u,v),\\ \,\,\,\,y+\Delta {s}_{y}^{(i)}(u,v),u,v),{H}_{i}(x,y,u,v)))\\ (\Delta {s}_{x}^{(i)}(u,v),\Delta {s}_{y}^{(i)}(u,v))=Findmax(Corr({I}_{i}(x,y)\\ \,\,\,\,\otimes \,{H}_{i}(x,y,u,v),{V}_{h}(x,y,u,v)))\\ {\phi }_{i+1}(u,v)={\phi }_{i}(u,v)\,if\,i\,mod\,3 > 0\\ {\phi }_{i+1}(u,v)={\phi }_{i}(u,v)+c\times \iint (\Delta {s}_{x}^{(i)}(u,v),\\ \,\,\,\,\Delta {s}_{y}^{(i)}(u,v))dudv\,if\,i\,mod\,3=0\\ \begin{array}{c}{\phi }_{1}(u,v)=0\,or\,{\phi }_{system}(u,v)\\ (\Delta {s}_{x}^{(1)}(u,v),\Delta {s}_{y}^{(1)}(u,v))=(0,\,0)\end{array}\end{array}$$

where *I*_*i*_(*x*, *y*) is the intermediate reconstruction image of the *i*th iteration, *N*_angle_ is the total number of the effective angles determined by the pupil shape, and *H*_*i*_(*x*, *y*, *u*, *v*) is the PSF for the view (*u*, *v*) generated with the estimated aberration *A*(*u*, *v*)exp(j*φ*_*i*_(*u*, *v*)) added in the pupil plane. The PSF generation function PSFGen() is described as before based on the Rayleigh–Sommerfeld transfer function^57^. Deconv(*A*, *B*) is the deconvolution of image *A* by the kernel of *B* with the state-of-the-art fast deconvolution method based on the hyper-Laplacian prior^58^. Corr() represents the calculation of the cross-correlation matrix in the spatial domain to estimate the lateral shifts with the function of Findmax() to find the coordinates of the peak value. For the initial value of the aberrated wavefront *φ*_1_(*u*, *v*), we can either set it to zero or the local system aberration *φ*_system_(*u*, *v*) calibrated before. In previous DAO in geometric optics^33^, we simplify the influence of the aberration as lateral shifts of the ideal angular PSFs *H*_*i*_(*x*, *y*, *u*, *v*). However, we find that the aberration will also influence the additional coherence among different views introduced by the coded MLA, leading to changes of the PSF distributions. Therefore, we extend the DAO in wave-optics model by updating the aberrated PSFs with estimated aberrations based on the wave-optics model for each three iterations during the ADMM method (Extended Data Fig. 3b). We use the Zernike polynomial up to order 45 to fit the aberrated phase during iterations. For the convergence of iteration, we set the threshold as 0.1 wavelength in terms of the root mean square (r.m.s.) of the residual phase. It usually takes about two iterations to converge for the additional wavefront with an r.m.s. of 1 wavelength, and ten iterations for the additional wavefront with an r.m.s. of 5 wavelengths. The wavefront estimation process takes approximately 32 s for 1 iteration on 5 × 5 scanning light-field images with a pixel number of 2,000 × 2,000 on a desktop computer (CPU, Intel i9-10940X; RAM, 128 GB; GPU, NVIDIA GeForce RTX Titan).

Finally, the ISA with DAO for each local region is obtained by phase-space deconvolution with the estimated aberrated PSFs. It usually takes approximately 9 s for 5 × 5 scanning light-field images with a pixel number of 2,000 × 2,000 on a desktop computer with a graphical processing unit with specifications as listed previously.

### Numerical simulation

A series of numerical simulations were conducted to verify the performance of ISA and DAO quantitatively. The simulated measurements were generated by convolution of the ground-truth images with the simulated PSFs. The ground-truth images used in Extended Data Figs. 1, 2, 4, 5 were captured by a conventional sensor with a high-quality camera lens. The parameters of the simulated imaging process were the same as the experimental imaging system with a centre wavelength of 525 nm, a magnification factor of 10, and the imaging *f*-number at the objective plane of 1 (corresponding to an *f*-number of 10 at the image plane). For the simulation of the PSF and optical transfer function, we chose the centre wavelength of 1,000 nm for simplification (Extended Data Fig. 1). Gaussian noises were added to simulate the readout noise of the CMOS sensor. We used both peak signal-to-noise ratio (SNR) and SSIM to evaluate the reconstruction results. To characterize the performance of DAO (Extended Data Figs. 4, 5), we added different levels of aberrations at the pupil plane in terms of r.m.s. with a maximum Zernike order of 45. The ratio *σ* between the r.m.s. of the residual wavefront error and the peak–valley value of the ground-truth aberrated wavefront was used to show the performance of wavefront correction. We assumed that the hardware adaptive optics could measure the aberrated wavefronts perfectly and correct the aberration with a state-of-the-art tip-tilt-based deformable mirror array of 15 × 15 segments, corresponding to the same view number of the meta-imaging sensor. The simulated results of hardware adaptive optics were obtained by convolution of the ground-truth image with the wide-field PSF generated by the residual pupil wavefront, and the simulated results of a conventional 2D sensor were obtained by convolution of the ground-truth image with the wide-field PSF generated by the aberrated pupil wavefront.

### Learning-based reconstruction

Deep neural networks can be applied to further enhance the imaging performance of the meta-imaging sensor or accelerate the reconstruction process by exploiting the data prior. Although we used the physics-based optimization algorithms for all the experiments to characterize the performance of meta-imaging sensor, we have tested several existing neural networks with pretrained models to show their great potential for future development. For image super-resolution shown in Extended Data Fig. 10a–c, we applied two single image super-resolution networks^47,48^ with their respective open-source pre-trained models. We used the reconstructed results of the meta-imaging sensor directly as the input of these networks to show that the meta-imaging sensor is also compatible with existing imaging processing algorithms to further improve the performance using a data prior. For the acceleration of the motion correction process shown in Extended Data Fig. 10d–g, we used the recurrent back-projection network^49^ with its open source pre-trained model. We utilized the 3 × 3 low-resolution images of the centre view as the input of the network to obtain the high-resolution image without motion artefacts. Although the output of the network has slightly reduced spatial resolution compared with the results obtained by our optical-flow-based motion correction algorithm, the inference of the network is about 29 times faster than the optimization algorithm, demonstrating the potential of applying deep neural networks to accelerate all the reconstruction processes of the meta-imaging sensor.

### Depth estimation

The disparities between different views provide clues for depth sensing based on multiview stereo^59^. The meta-imaging sensor captures the spatial–angular measurements with better resolution than that of a traditional light-field camera, facilitating high-density depth sensing with better accuracy. To show the improvement, we applied the same depth estimation method on the data of traditional light field and the meta-imaging sensor separately. We chose a state-of-the-art depth estimation algorithm designed for light field with super-pixel-based regularization over partially occluded regions^42^. The disparity map obtained by this method was converted into actual distances or thickness on the basis of the geometric parameters of the imaging system. To evaluate the accuracy of the depth estimation, we used a commercial focus-variation microscope (InfiniteFocus G5plus, Bruker Alicona)^43^ to measure the ground truth of the depth map for the circuit board in Fig. 6. It takes about half an hour to capture the whole FOV through scanning over a large volume for the commercial system. Finally, the thickness errors were calculated after accurate alignment between the reconstruction results of different methods and the ground truth. More importantly, we find that the optical aberrations will influence the accuracy of the depth map as well for vision-based depth sensing, which are hard to correct in traditional imaging systems. Here we used the aberration maps obtained by initial global aberration estimation to warp different views for geometric correction before depth estimation. Results with aberration correction show better performance than those without correction (Extended Data Fig. 9c–f).

## Online content

Any methods, additional references, Nature Research reporting summaries, source data, extended data, supplementary information, acknowledgements, peer review information; details of author contributions and competing interests; and statements of data and code availability are available at 10.1038/s41586-022-05306-8.

## Supplementary information

Supplementary Information The file contains Supplementary Table 1, which gives the experimental parameters used for the data acquisition and reconstruction of the meta-imaging sensor.

Supplementary Video 1 Imaging through strong nonuniform aberrations introduced by multiple layers of plastic wrap.

Supplementary Video 2 The pipeline and demonstration of the motion correction algorithm for dynamic scenes.

Supplementary Video 3 Multisite digital adaptive optics (DAO) for the ground-based 80-cm telescope.

Supplementary Video 4 Depth sensing for the application of autonomous driving.

Supplementary Video 5 Depth sensing for the application of industrial inspection.

## Data Availability

All data generated or analysed during this study are included in this published article and the Zenodo repository with the following links: 10.5281/zenodo.6641847, 10.5281/zenodo.6643915 and 10.5281/zenodo.6644095. Source data are provided with this paper.

## Source data

Source Data Fig. 2
Source Data Fig. 3
Source Data Fig. 5
Source Data Fig. 6
Source Data Extended Data Fig. 4
Source Data Extended Data Fig. 5
Source Data Extended Data Fig. 6

## Code availability

The codes for the whole pipeline of the meta-imaging sensor are available from https://github.com/freemercury/Aberration_correction_metasensor.

## References

[1] Yurtsever E, Lambert J, Carballo A, Takeda K (2020). A survey of autonomous driving: common practices and emerging technologies. IEEE Access.

[2] Brady DJ (2012). Multiscale gigapixel photography. Nature.

[3] Yun SH, Kwok SJJ (2017). Light in diagnosis, therapy and surgery. Nat. Biomed. Eng..

[4] Lichtman JW, Conchello J-A (2005). Fluorescence microscopy. Nat. Methods..

[5] Hardy, J. W. *Adaptive Optics for Astronomical Telescopes* (Oxford Univ. Press, 1998).

[6] Cossairt OS, Miau D, Nayar SK (2011). Scaling law for computational imaging using spherical optics. J. Opt. Soc. Am. A.

[7] Sasián, J. *Introduction to Aberrations in Optical Imaging Systems* (Cambridge Univ. Press, 2013).

[8] Roggemann, M. C. & Welsh, B. M. *Imaging through Turbulence* (CRC, 2018).

[9] Rogers C (2021). A universal 3D imaging sensor on a silicon photonics platform. Nature.

[10] Conrady, A. E. *Applied Optics and Optical Design, Part One* (Courier, 2013).

[11] Lohmann AW, Dorsch RG, Mendlovic D, Zalevsky Z, Ferreira C (1996). Space–bandwidth product of optical signals and systems. J. Opt. Soc. Am. A.

[12] Sutherland W (2015). The Visible and Infrared Survey Telescope for Astronomy (VISTA): design, technical overview, and performance. Astron. Astrophys..

[13] Fan J (2019). Video-rate imaging of biological dynamics at centimetre scale and micrometre resolution. Nat. Photonics.

[14] McConnell G (2016). A novel optical microscope for imaging large embryos and tissue volumes with sub-cellular resolution throughout. eLife.

[15] Wu R (2018). Design of freeform illumination optics. Laser Photonics Rev..

[16] Khorasaninejad M (2016). Metalenses at visible wavelengths: diffraction-limited focusing and subwavelength resolution imaging. Science.

[17] Xu, L. & Jia, J. Two-phase kernel estimation for robust motion deblurring. In *Proc. European Conf. Computer Vision (ECCV)* (eds Daniilidis, K. et al.) 157–170 (2010).

[18] Heide F (2013). High-quality computational imaging through simple lenses. ACM Trans. Graph..

[19] Schuler, C. J., Hirsch, M., Harmeling, S. & Schölkopf, B. Blind correction of optical aberrations. In *Proc. European Conf. Computer Vision (ECCV)* (eds Fitzgibbon, A. et al.) 187–200 (2012).

[20] Chung J, Martinez GW, Lencioni KC, Sadda SR, Yang C (2019). Computational aberration compensation by coded-aperture-based correction of aberration obtained from optical Fourier coding and blur estimation. Optica.

[21] Peng Y (2019). Learned large field-of-view imaging with thin-plate optics. ACM Trans. Graph..

[22] Koh J, Lee J, Yoon S (2021). Single-image deblurring with neural networks: a comparative survey. Comput. Vis. Image Underst..

[23] Booth MJ (2007). Adaptive optics in microscopy. Philos. Trans. R. Soc., A.

[24] Booth MJ (2014). Adaptive optical microscopy: the ongoing quest for a perfect image. Light: Sci. Appl..

[25] Wizinowich P (2000). First light adaptive optics images from the Keck II telescope: a new era of high angular resolution imagery. Publ. Astron. Soc. Pac..

[26] Ivezić Ž (2019). LSST: from science drivers to reference design and anticipated data products. Astrophys. J..

[27] Adelson EH, Wang JYA (1992). Single lens stereo with a plenoptic camera. IEEE Trans. Pattern Anal. Mach. Intell..

[28] Levoy, M. & Hanrahan, P. Light field rendering. In *Proc. 23rd Annual Conf. Computer Graphics and Interactive Techniques* (ed. Fujii, J.) 31–42 (ACM, 1996).

[29] Ng, R. et al. *Light Field Photography with a Hand-held Plenoptic Camera*. Stanford University Computer Science Tech Report CSTR 2005-02 (Stanford Univ., 2005).

[30] Ng, R. & Hanrahan, P. M. Digital correction of lens aberrations in light field photography. In *Proc. Intl Optical Design Conf.* WB2 (OSA, 2006).

[31] Levoy, M., Ng, R., Adams, A., Footer, M. & Horowitz, M. Light field microscopy. *ACM Trans. Graph.* **25**, 924–934 (2006).

[32] Ihrke I, Restrepo J, Mignard-Debise L (2016). Principles of light field imaging: briefly revisiting 25 years of research. IEEE Signal Process. Mag..

[33] Wu, J. et al. Iterative tomography with digital adaptive optics permits hour-long intravital observation of 3D subcellular dynamics at millisecond scale. *Cell* **184**, 3318-3332.e17 (2021). 10.1016/j.cell.2021.04.029 34038702

[34] Milkie DE, Betzig E, Ji N (2011). Pupil-segmentation-based adaptive optical microscopy with full-pupil illumination. Opt. Lett..

[35] Dong, J., Pan, J., Su, Z. & Yang, M.-H. Blind image deblurring with outlier handling. In *Proc. IEEE Intl Conf. Computer Vision (ICCV)* (eds Ikeuchi, K. et al.) 2497–2505 (IEEE, 2017).

[36] Jin, M., Roth, S. & Favaro, P. Normalized blind deconvolution. In *Proc. European Conf. Computer Vision (ECCV)* (eds Ferrari, V. et al.) 694–711 (2018).

[37] Liu, Y., Dong, W., Gong, D., Zhang, L. & Shi, Q. Deblurring natural image using super-Gaussian fields. In *Proc. European Conf. Computer Vision (ECCV)* (eds Ferrari, V. et al.) 467–484 (2018).

[38] Bai Y, Cheung G, Liu X, Gao W (2018). Graph-based blind image deblurring from a single photograph. IEEE Trans. Image Process..

[39] Yue, T., Suo, J., Wang, J., Cao, X. & Dai, Q. Blind optical aberration correction by exploring geometric and visual priors. In *Proc. IEEE Conf. Computer Vision and Pattern Recognition (CVPR)* 1684–1692 (IEEE, 2015).

[40] Robertson BE (2019). Galaxy formation and evolution science in the era of the Large Synoptic Survey Telescope. Nat. Rev. Phys..

[41] Deng L (2021). Lenghu on the Tibetan Plateau as an astronomical observing site. Nature.

[42] Chen J, Hou J, Ni Y, Chau LP (2018). Accurate light field depth estimation with superpixel regularization over partially occluded regions. IEEE Trans. Image Process..

[43] Zangl K, Danzl R, Helmli F, Prantl M (2018). Highly accurate optical μCMM for measurement of micro holes. Procedia CIRP.

[44] Chen Z (2021). Electron ptychography achieves atomic-resolution limits set by lattice vibrations. Science.

[45] Zheng G, Horstmeyer R, Yang C (2013). Wide-field, high-resolution Fourier ptychographic microscopy. Nat. Photonics..

[46] Zhu S, Lai A, Eaton K, Jin P, Gao L (2018). On the fundamental comparison between unfocused and focused light field cameras. Appl. Opt..

[47] Lim, B., Son, S., Kim, H., Nah, S. & Lee, K. M. Enhanced deep residual networks for single image super-resolution. In *Proc. IEEE Conf. Computer Vision and Pattern Recognition (CVPR Workshops)* 1132–1140 (IEEE, 2017).

[48] Wang, X. et al. ESRGAN: Enhanced Super-Resolution Generative Adversarial Networks. In *Proc. European Conf. Computer Vision (ECCV Workshops)*(eds Leal-Taixé, L. & Roth, S.) 63–79 (2018).

[49] Haris, M., Shakhnarovich, G., & Ukita, N. Recurrent back-projection network for video super-resolution. In *Proc.**IEEE Conf. Computer Vision and Pattern Recognition (CVPR)* 3892–3901 (IEEE, 2019).

[50] Wu J (2016). Snapshot hyperspectral volumetric microscopy. Sci. Rep..

[51] Liu, C. *Beyond Pixels: Exploring New Representations and Applications for Motion Analysis*. PhD thesis, Massachusetts Institute of Technology (2009).

[52] Gallagher RH (1986). Finite element structural analysis. Int. J. Numer. Methods Eng..

[53] Watson, D. F. *Contouring: A Guide to the Analysis and Display of Spatial Data* (Elsevier, 2013).

[54] Gustafsson MG (2005). Nonlinear structured-illumination microscopy: wide-field fluorescence imaging with theoretically unlimited resolution. Proc. Natl. Acad. Sci..

[55] Lu Z (2019). Phase-space deconvolution for light field microscopy. Opt. Express.

[56] Boyd S, Parikh N, Chu E, Peleato B, Eckstein J (2011). Distributed optimization and statistical learning via the alternating direction method of multipliers. Found. Trends Mach. Learn..

[57] Zhang Y (2021). Computational optical sectioning with an incoherent multiscale scattering model for light-field microscopy. Nat. Commun..

[58] Krishnan D, Fergus R (2009). Fast image deconvolution using hyper-Laplacian priors. Adv. Neural Inf. Process. Syst..

[59] Zhang Y (2017). Light-field depth estimation via epipolar plane image analysis and locally linear embedding. IEEE Trans. Circuits Syst. Video Technol..

