# Supplementary material for: An integrated imaging sensor for aberration-corrected 3D photography
Source: Nature. 2022 Oct 19;612(7938):62–71. doi: 10.1038/s41586-022-05306-8 (PMC9712118; doi:10.1038/s41586-022-05306-8)
Supplement: Supplementary file 1 — The file contains Supplementary Table 1, which gives the experimental parameters used for the data acquisition and reconstruction of the meta-imaging sensor. [file 41586_2022_5306_MOESM1_ESM.pdf]

nature portfolio

https://doi.org/10.1038/s41586-022-05306-8

Supplementary information

An integrated imaging sensor for aberration-corrected 3D photography

In the format provided by the
authors and unedited

Supplementary information

An integrated imaging sensor for aberration-corrected 3D photography

Jiamin Wu[*], Yuduo Guo[*], Chao Deng[*], Anke Zhang, Hui Qiao, Zhi Lu, Jiachen Xie, Lu Fang[†] & Qionghai Dai[†]

[*]*These authors contributed equally to this work*

[†]Correspondences: qhdai@tsinghua.edu.cn (Q. D.), fanglu@tsinghua.edu.cn (L. F.)

Supplementary Table 1| Experimental parameters

<table>
<tr><th></th><th>Lens type</th><th>Lens parameter (Focal length/ effective F-number)</th><th>Frame rate</th><th>Exposure time</th><th>Scanning period</th><th>Block size (sensor pixels)</th><th>Motion correction</th></tr>
<tr><td>1f, ED10a-c</td><td>Plastic lens</td><td>135 mm/10</td><td>30 Hz</td><td>1.5 ms</td><td>5×5</td><td>251×251</td><td>off</td></tr>
<tr><td>2</td><td rowspan="2">Plastic lens</td><td rowspan="2">135 mm/10</td><td rowspan="2">30 Hz</td><td rowspan="2">11 ms</td><td rowspan="2">5×5</td><td>351×351</td><td>off</td></tr>
<tr><td>ED6a-e</td><td>333×333</td><td>off</td></tr>
<tr><td rowspan="2">ED6f-i</td><td>Canon EF 70-200mm 1:2.8L</td><td rowspan="2">135 mm/10</td><td rowspan="2">30 Hz</td><td rowspan="2">11 ms</td><td rowspan="2">5×5</td><td rowspan="2">351×351</td><td>off</td></tr>
<tr><td>Plastic lens</td><td>off</td></tr>
<tr><td>ED7c</td><td rowspan="2">Thorlabs LA1725-A</td><td rowspan="2">400 mm/10</td><td rowspan="2">10 Hz</td><td rowspan="2">75 ms</td><td rowspan="2">5×5</td><td>1501×1501</td><td>off</td></tr>
<tr><td>ED7a-g</td><td>1301×1301</td><td>off</td></tr>
<tr><td>3b-g, i, j SV1</td><td>Canon EF 300mm 1:2.8L</td><td>300 mm/10</td><td>30 Hz</td><td>75 ms</td><td>5×5</td><td>201×201</td><td>off</td></tr>
<tr><td>4, SV2</td><td>Plastic lens</td><td>135 mm/10</td><td>30 Hz</td><td>1.5 ms</td><td>5×5</td><td>801×801</td><td>on</td></tr>
<tr><td>5a-b, g, SV3, ED10a-c</td><td rowspan="2">Tsinghua-NAOC 80-cm telescope</td><td rowspan="2">8000 mm/10</td><td rowspan="2">15 Hz</td><td rowspan="2">2 ms</td><td rowspan="2">5×5</td><td>251×251</td><td>on</td></tr>
<tr><td>5c-e</td><td>151×151</td><td>on</td></tr>
<tr><td>6a, c, SV4</td><td>Canon EF 50mm 1:1.2L</td><td>50 mm/10</td><td>30 Hz</td><td>2.5 ms</td><td>5×5</td><td>-</td><td>off</td></tr>
<tr><td>6e, ED9, SV5</td><td>Olympus MV PLAPO 0.63×</td><td>1.8× magnification / 0.15NA</td><td>15 Hz</td><td>35 ms</td><td>5×5</td><td>-</td><td>off</td></tr>
<tr><td>ED8b, d-g</td><td>Tsinghua-NAOC 80-cm Cassegrain telescope</td><td>8000 mm/10</td><td>15 Hz</td><td>2 ms</td><td>5×5</td><td>251×251</td><td>on</td></tr>
<tr><td>ED10d-f</td><td>Canon EF 50mm 1:1.2L</td><td>50 mm/10</td><td>30 Hz</td><td>30 ms</td><td>3×3</td><td>708×930</td><td>on</td></tr>
</table>
